# Integrated analysis of 454 and Illumina transcriptomic sequencing characterizes carbon flux and energy source for fatty acid synthesis in developing *Lindera glauca* fruits for woody biodiesel

**DOI:** 10.1186/s13068-017-0820-2

**Published:** 2017-05-25

**Authors:** Zixin Lin, Jiyong An, Jia Wang, Jun Niu, Chao Ma, Libing Wang, Guanshen Yuan, Lingling Shi, Lili Liu, Jinsong Zhang, Zhixiang Zhang, Ji Qi, Shanzhi Lin

**Affiliations:** 1 Beijing Advanced Innovation Center for Tree Breeding by Molecular Design, College of Biological Sciences and Biotechnology, College of Nature Conservation, National Engineering Laboratory for Tree Breeding, Key Laboratory of Genetics and Breeding in Forest Trees and Ornamental Plants, Ministry of Education, Beijing Forestry University, Beijing, 10083 China; 2 College of Horticulture and Landscape Architecture, Key Laboratory of Protection and Development Utilization of Tropical Crop Germplasm Resources, Ministry of Education, Hainan University, Haikou, 570228 China; 3 Research Institute of Forestry, Chinese Academy of Forestry, Beijing, 10091 China

**Keywords:** *Lindera glauca* fruits, Woody biodiesel, Oil synthesis, Illumina and 454 sequencing, Carbon flux and energy source, Differential expression profiles

## Abstract

**Background:**

*Lindera glauca* fruit with high quality and quantity of oil has emerged as a novel potential source of biodiesel in China, but the molecular regulatory mechanism of carbon flux and energy source for oil biosynthesis in developing fruits is still unknown. To better develop fruit oils of *L. glauca* as woody biodiesel, a combination of two different sequencing platforms (454 and Illumina) and qRT-PCR analysis was used to define a minimal reference transcriptome of developing *L. glauca* fruits, and to construct carbon and energy metabolic model for regulation of carbon partitioning and energy supply for FA biosynthesis and oil accumulation.

**Results:**

We first analyzed the dynamic patterns of growth tendency, oil content, FA compositions, biodiesel properties, and the contents of ATP and pyridine nucleotide of *L. glauca* fruits from seven different developing stages. Comprehensive characterization of transcriptome of the developing *L. glauca* fruit was performed using a combination of two different next-generation sequencing platforms, of which three representative fruit samples (50, 125, and 150 DAF) and one mixed sample from seven developing stages were selected for Illumina and 454 sequencing, respectively. The unigenes separately obtained from long and short reads (201, and 259, respectively, in total) were reconciled using TGICL software, resulting in a total of 60,031 unigenes (mean length = 1061.95 bp) to describe a transcriptome for developing *L. glauca* fruits. Notably, 198 genes were annotated for photosynthesis, sucrose cleavage, carbon allocation, metabolite transport, acetyl-CoA formation, oil synthesis, and energy metabolism, among which some specific transporters, transcription factors, and enzymes were identified to be implicated in carbon partitioning and energy source for oil synthesis by an integrated analysis of transcriptomic sequencing and qRT-PCR. Importantly, the carbon and energy metabolic model was well established for oil biosynthesis of developing *L. glauca* fruits, which could help to reveal the molecular regulatory mechanism of the increased oil production in developing fruits.

**Conclusions:**

This study presents for the first time the application of an integrated two different sequencing analyses (Illumina and 454) and qRT-PCR detection to define a minimal reference transcriptome for developing *L. glauca* fruits, and to elucidate the molecular regulatory mechanism of carbon flux control and energy provision for oil synthesis. Our results will provide a valuable resource for future fundamental and applied research on the woody biodiesel plants.

**Electronic supplementary material:**

The online version of this article (doi:10.1186/s13068-017-0820-2) contains supplementary material, which is available to authorized users.

## Background

Biodiesel, an alternative diesel fuel, has been identified as an environment-friendly fuel for its biodegradability, low-emissions, and renewability. However, the biodiesel presents a significant challenge because of high-cost feedstock and increasingly aggravating tension between energy crisis and food security [1]. In recent years, seed oils of woody plants (such as *Prunus sibirica*, *Xanthoceras sorbifolia*, *Pistacia chinensis,* and *Jatropha curcas*) with an obvious advantage over conventional feedstocks have been used extensively as the raw materials for biodiesel production in China [2–4]. Thus, it is necessary to develop the non-food plant resources for biodiesel, especially in China with large population and low per-capita arable land.

*Lindera glauca*, small arbor deciduous tree of the family Lauraceae and the genus *Lindera Thunb*, is widely distributed in the mountainous districts at low altitudes only in China, Japan, and Korea [5, 6]. In China, this plant is one of the most ecologically and economically important tree species owing to its plentiful resource, superior adaptability, ecological benefits, and medicinal utilization [5, 6]. Early studies in different germplasms of *L. glauca* have shown that the oil content of the ripened seeds, ranged from 42.0 to 53.0% [5, 7, 8], which was higher than that of traditional oil plants [9]. It was estimated that the annual yields of *L. glauca* fruits and seeds are greater than 100,000 and 22,200 tons, and the average productions of ripened fruits and seeds are about 11.5 and 2.5 tons/ha in China, respectively [5, 10]. In general, the oils of *L. glauca* fruits or seeds have been used as an edible oil or important raw material for daily-use chemical products (such as soap, detergent, cosmetics, surfactants, and lubricants) [5]. Presently, based on the evaluation of oil content, FA composition, and physicochemical properties in 74 samples from 9 genera and 47 species of Lauraceae, *L. glauca* has been selected as non-food plant resource for biodiesel [11]. Importantly, according to our studies on 102 *L. glauca* fruit samples from nine geographical provenances, seven wild germplasm accessions have been identified with rich oil content and a high percentage of oleic and linoleic acid [10, 12]. All these indicated that *L. glauca* fruit oils may be useful as a novel potential source of biodiesel feedstock in China. However, the molecular regulatory mechanism of oil accumulation in developing *L. glauca* fruits is still very poorly understood, and the nature of carbon flux control and energy provision remains one of the most interesting open challenges encountered in the study of FA biosynthesis. Thus, understanding the molecular basis of oil biosynthesis in developing *L. glauca* fruits has become an imperative for the development of woody biodiesel.

The de novo FA biosynthesis, localized in plastids of plants, requires acetyl-CoA, ATP, and reducing power [13]. There exist different pathways in cellular metabolism responsible for allocating carbon source, reducing power, and energy required for FA biosynthesis in plants [14]. Heterotrophic sink organs (such as developing fruits, seeds, and roots) are supplied with carbon source and energy mostly as sucrose from photosynthetic tissues [15]. The channeling of sucrose into metabolism requires its cleavage by several isoforms of sucrose synthase (SUS) and invertase (INV) localized in different subcellular compartments [16, 17], and the resulting product is converted to pyruvate (PYR) via the glycolysis or to glyceraldehyde 3-phosphate (GAP) through oxidative pentose phosphate pathway (OPPP) in both cytosol and plastid [13, 18]. Many studies have shown that a broad range of metabolites can be utilized by plastids as carbon source for FA biosynthesis [13, 19–24], but almost all of which are based on studies of ability of isolated plastids to incorporate exogenous metabolites into FAs. Moreover, the relative rates of utilizations of exogenous metabolites for FA biosynthesis could also vary due to the regulation of selective plastidial transporter [13, 25–27], mainly including glycolipid transporter (GLT), glucose-6-phosphate transporter (GPT), phosphoenolpyruvate transporter (PPT), xylulose 5-phosphate/phosphate translocator (XPT), and triose phosphate transporter (TPT). However, the majority of the transporters have not yet been characterized at the molecular level. Also noteworthy was the impermeability of biomembranes to acetyl-CoA [28], and therefore supply of acetyl-CoA for de novo FA synthesis in plastids and FA elongation in cytosol must be synthesized within each compartment by alternative enzymes of PYR dehydrogenase complex (PDC), ATP-citrate lyase (ACL), acetyl-CoA synthetase (ACS), or carnitine acetyltransferase (CA) [28, 29]. All these findings have shown one complexity of carbon flux allocation into FA synthesis in plants. Hence, it is needed to unravel the mechanisms of carbon flux distribution and control for FA synthesis destined to oil accumulation in plants.

In addition to carbon supply, de novo FA synthesis has a high demand for ATP and reducing power (in the form of NADH and NADPH). It is known that ATP is required for the carboxylation of acetyl-CoA to malonyl-CoA by acetyl-CoA carboxylase (ACC) complex, while NADPH and NADH are required in the steps of 3-ketoacyl-ACP2 reductase (KAR) and 2-enoyl-ACP reductase (EAR), respectively [30]. ATP is generally supplied by mitochondrial oxidative phosphorylation, chloroplast photophosphorylation, or substrate-level phosphorylation in glycolysis. The reducing power may be generated as a result of glycolysis, tricarboxylic acid (TCA) cycle, and PDC activity as NADH, or by the metabolism via OPPP and NADP-dependent malic enzyme (NADP-ME) reaction which generates NADPH [13]. However, little attention has been paid to the sources of ATP and reducing power for FA synthesis. Thus, determining the potential energy source for FA synthesis in developing *L. glauca* fruits is important.

In recent years, Illumina sequencing, one next-generation sequencing (NGS) technology, has been applied for the transcriptional expression studies in many oil plants [16, 31–38], but some short sequences are not so effective to get BLAST hits owing to lack of a characterized protein domain [32, 39]. The recently established 454 sequencing platform can provide the longer reads, and has been used to generate well-defined transcriptomes in many oil plants, such as Siberian apricot, oilseed rape, olive, and peanut [39–43]. Importantly, a combination of 454 and Illumina sequencing has been used to define a minimal reference transcriptome for maritime pine [44] and globe artichoke [45], which provides a powerful means to study gene function and regulation. Recently, we have performed 454 sequencing analysis for different tissues of *L. glauca* [46], but the obtained data are not still suitable for us to deeply explore regulatory mechanisms of oil accumulation in developing fruits.

In this sequential study, we performed an integrated analysis of two different NGS platforms (Illumina and 454) and qRT-PCR detection as an important attempt to assess the molecular regulatory mechanism of the provision of carbon flux and energy for oil biosynthesis in developing *L. glauca* fruits. We first detected the dynamic patterns of growth tendency (weight and size) and oil accumulation (content and composition) as well as the contents of ATP and reducing power (NADH and NADPH) at seven different developing stages (25, 50, 75, 100, 125, 150, and 175 DAF) of fruits, and then biodiesel properties of oils from developing fruits were evaluated. As a result, three representative fruit samples at 50, 125, and 150 DAF and one mixed sample from seven developing stages were selected for Illumina and 454 sequencing, respectively. The long and short reads separately obtained from 454 and Illumina sequencing was assembled using Trinity software, and then the differentially expressed genes were screened. To define a minimal reference transcriptome for developing *L. glauca* fruits, all the unigenes obtained separately from long and short reads were reconciled by means of TGICL software, and then were functionally annotated. Finally, some key genes involved specifically in photosynthesis, sucrose cleavage, glycolysis, OPPP, TCA cycle, metabolite transport, acetyl-CoA formation, FA biosynthesis, and TAG assembly as well as the source of ATP and reducing power, were characterized by means of a combination of 454 and Illumina sequencing as well as qRT-PCR analysis. Our findings provide new insights into the molecular regulatory mechanism of carbon flux allocation and energy source for oil accumulation in developing *L. glauca* fruits for the development of woody biodiesel.

## Results

### Dynamic patterns of growth and oil accumulation of developing fruits

**Fig. 1:**
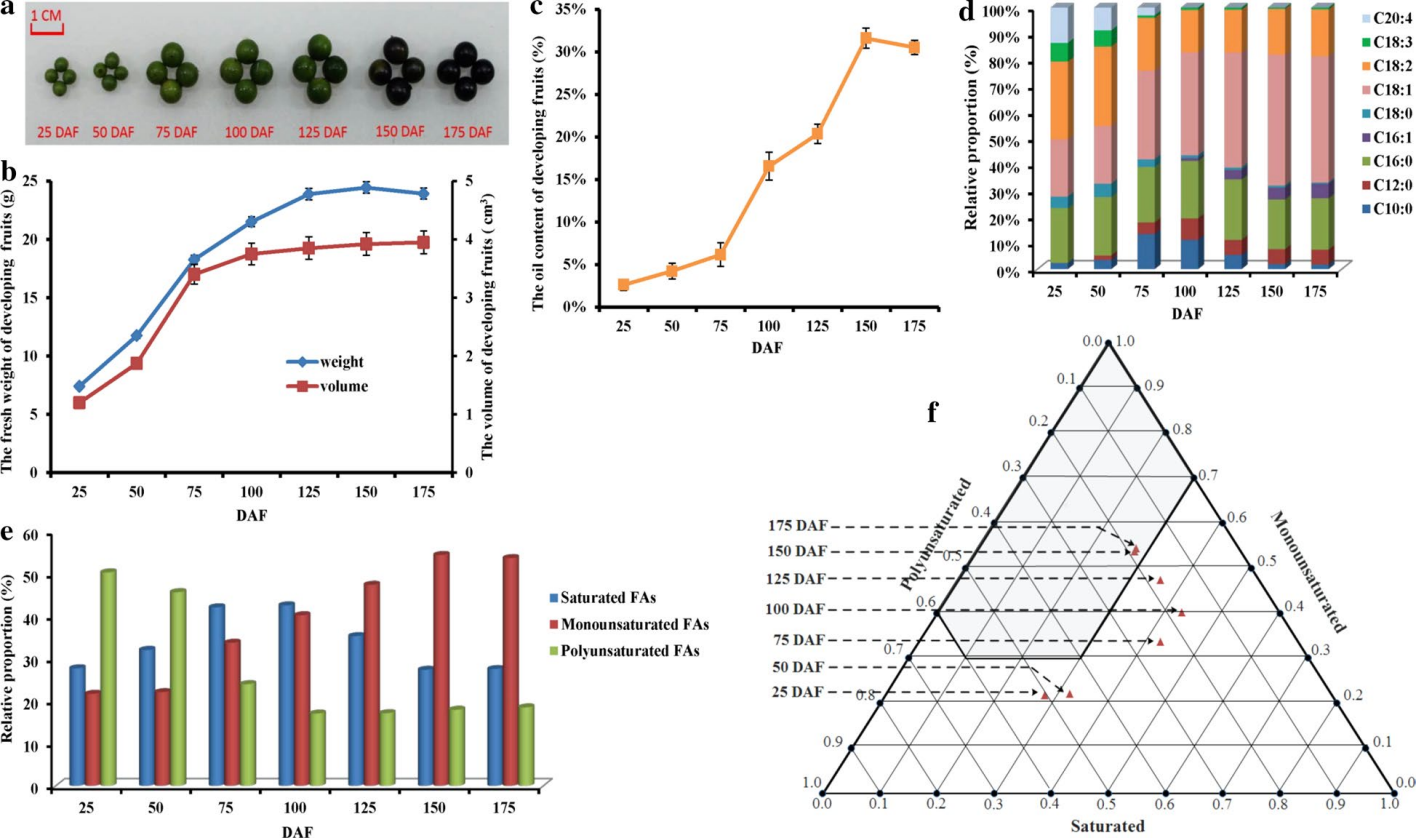
Dynamic changes of oil accumulation and biodiesel fuel properties in developing *L. glauca* fruits. **a** The feature of *L. glauca* fruits from seven developmental stages. **b** The growth tendency of *L. glauca* fruits during development. **c** The oil contents of *L. glauca* fruits at different developing stages. **d** Changes in the fatty acid (FA) compositions during fruit development. **e** The relative proportion of monounsaturated, polyunsaturated, and saturated FAs in developing *L. glauca* fruits. **f** Prediction chart of FA composition on biodiesel properties. The *gray part* of the region was clearly delineated to predict the biodiesel fuel properties, taking into account the cetane number, iodine number, cold filter plugging point, and oxidation-stability requirements. *Error bars* are standard deviations (SDs) of three biological replicates

To explore whether or not the growth and FA biosynthesis of *L. glauca* fruits responded to different developing stages, we analyzed the dynamic patterns of growth tendency (weight and size) and oil accumulation (oil content and FA composition) of the fruits during the whole developing stage from 25 DAF (immature stage) to 175 DAF (fully matured stage). We found that the weight of fruits was approximately 2.2-fold higher at 125 DAF than at 25 DAF, and 2.7% increase was observed at 150 DAF, followed by 1.7% decline at 175 DAF, which was in line with the temporal changes of fruit size during development (Fig. 1a, b), revealing that the development and growth of fruits was mainly at the early–middle stage (25–125 DAF). However, the oil content of developing fruits gradually increased with a rapid accumulation (about fivefold) from 6.16 ± 0.38% at 75 DAF to 31.62 ± 1.20% at 150 DAF and then slightly declined at 175 DAF (Fig. 1c), indicating an active oil accumulation of developing fruits at middle–late stage (75–150 DAF). Intriguingly, the oil content of fully ripened fruits, ranged from 30.54 to 31.62% (Fig. 1c), which was higher than that of traditional oilseed plants such as *S. sebiferum* (29.0%), *Phoebe sheareri* (24.4%), and *Lindera communis* (22.0%) [9, 11], implying a high quantity of oils for developing *L. glauca* fruits. In addition, by GC–MS analysis, we characterized nine kinds of FAs in the oils with differential temporal patterns of their relative proportions during fruit development (Table 1; Fig. 1d), among which C18:1 (oleic acid) was found as the most abundant compound, and its content increased from 25 to 175 DAF with a greater elevation at 50–150 DAF, while C18:2 (linoleic acid) and C18:3 (linolenic acid) exhibited a maximum value at 25–50 DAF and then declined, but remained relatively stable during later stages. Notably, C16:0 (palmitic acid) showed no significant alteration (19.05–23.22%) during the development, but C20:4 (arachidonic acid) and C16:1 (palmitoleic acid) were only detected at 25–75 DAF and 100–175 DAF, respectively (Table 1; Fig. 1d).

**Table 1:** The changes of FA compositions and their relative proportions during fruit development of *L. glauca*

DAF	C10:0 (%)	C12:0 (%)	C16:0 (%)	C16:1 (%)	C18:0 (%)	C18:1 (%)	C18:2 (%)	C18:3 (%)	C20:4 (%)
25	2.38 ± 0.14	–	21.02 ± 1.25	–	4.43 ± 0.43	21.79 ± 1.48	29.75 ± 1.55	7.16 ± 0.60	13.47 ± 1.12
50	3.57 ± 0.26	1.69 ± 0.11	21.61 ± 2.28	–	5.22 ± 0.40	22.19 ± 2.66	30.71 ± 2.10	6.21 ± 0.44	8.80 ± 1.26
75	13.49 ± 1.04	4.42 ± 0.29	21.23 ± 1.75	–	3.00 ± 0.19	33.78 ± 2.58	20.17 ± 1.30	0.96 ± 0.05	2.95 ± 0.11
100	11.22 ± 1.43	8.20 ± 0.54	22.06 ± 1.85	1.01 ± 0.09	1.13 ± 0.08	39.26 ± 2.87	16.27 ± 0.68	0.85 ± 0.04	–
125	5.44 ± 0.78	5.71 ± 0.51	23.22 ± 2.82	3.63 ± 0.12	0.97 ± 0.08	43.83 ± 2.64	16.41 ± 0.52	0.79 ± 0.05	–
150	1.96 ± 0.90	5.70 ± 0.47	19.05 ± 1.81	4.57 ± 0.05	0.78 ± 0.05	49.92 ± 2.36	17.57 ± 0.99	0.45 ± 0.04	–
175	1.79 ± 0.83	5.67 ± 0.34	19.73 ± 2.92	5.54 ± 0.11	0.47 ± 0.08	48.22 ± 1.84	17.95 ± 0.94	0.63 ± 0.05	–

Error bars are standard deviations (SD) of three biological replicates

### Evaluation of biodiesel fuel properties of oils from developing fruits

It is worth noticing that assessment on biodiesel fuel properties, such as iodine value (IV), cetane number (CN), cold filter plugging point (CFPP), and oxidation stability (OS), would provide the important references for the exploitation and utilization of biodiesel plants. Recently, a triangular prediction model of biodiesel fuel properties was constructed according to our studies on the influence of FA compositions from 10 woody biodiesel plants on fuel properties, emphasizing that the fuel properties of biodiesel product could be effectively predicted from FA compositions of raw material [47]. To determine the biodiesel properties of oils from developing *L. glauca* fruits, the percentages of monounsaturated, polyunsaturated, and saturated FAs in different developing fruits (Fig. 1e) were allocated into prediction model. The finding that the developing fruits at 150–175 DAF were located in the area (gray part) of our constructed triangular graph (Fig. 1f) indicated that the oils from *L. glauca* fruits at late developing stage, as a potential raw material for biodiesel, could completely meet the fuel properties. This was further supported by the fact that the average values of IV (86.0) and CN (54.0) of oils from late developing fruits satisfied the biodiesel standards of the USA (ASTM D6751, IN < 120, 47 < CN < 65) and European Organizations (EN 14214, IN < 120, 51 < CN < 65), but OS (4.3 h) and CFPP (−3.8 °C) only met the USA standard (3.0 h) and Germany standard in summer (DIN V 51606, <0 °C), respectively (Additional file 1: Table S1).

### Temporal change patterns of the contents of ATP and pyridine nucleotide in developing fruits

**Fig. 2:**
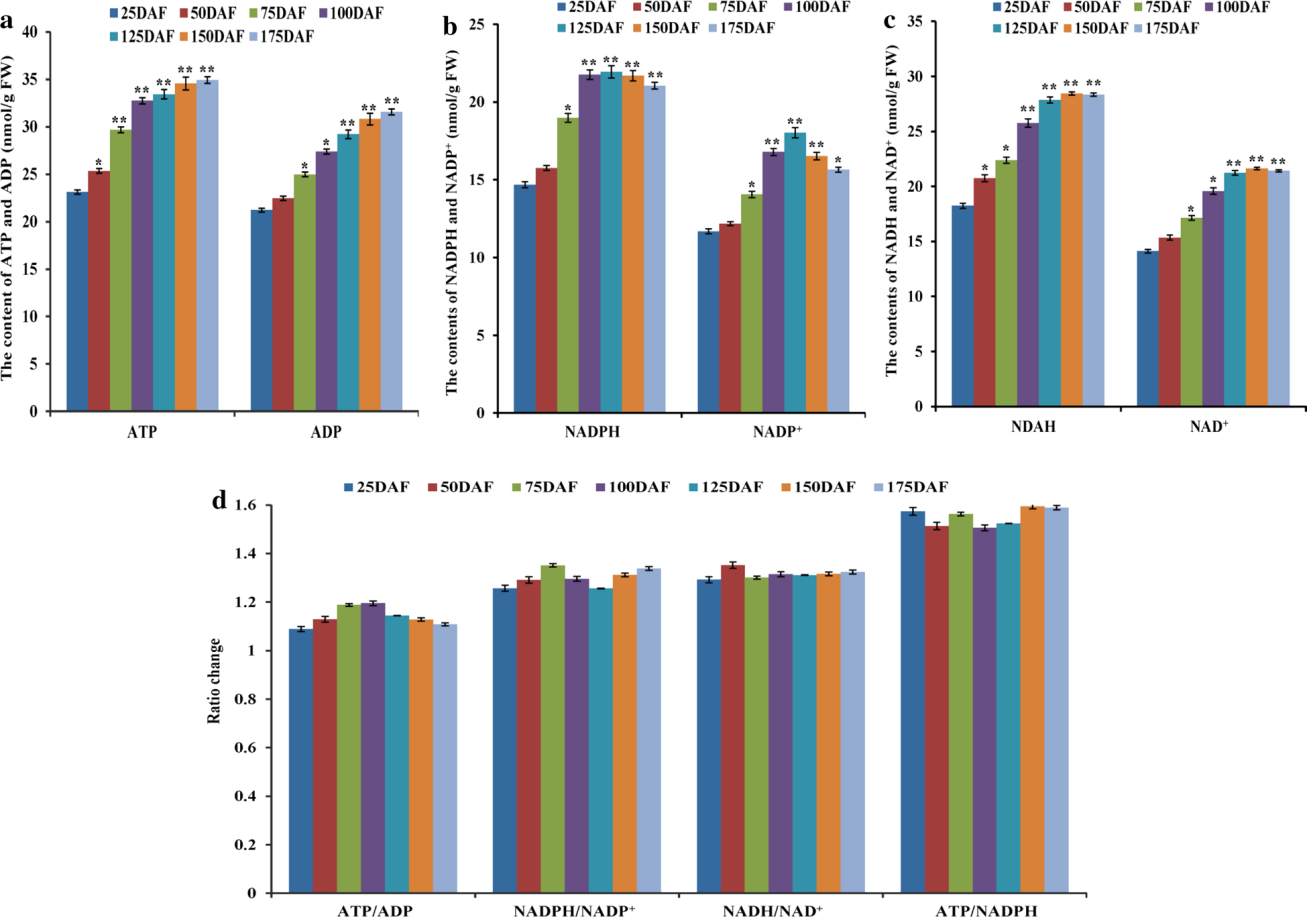
Temporal change analysis for ATP and pyridine nucleotide levels in developing *L. glauca* fruits. **a** ATP and ADP. **b** NADPH and NADP^+^. **c** NADH and NAD^+^. **d** The ratios of NADPH/NADP^+^, NADH/NAD^+^, ATP/ADP and ATP/NADPH. *Error bars* are SD of three biological replicates. *Asterisks* (*P < 0.05, **P < 0.01) indicate that the differences between 25 DAF and other developing stages are statistically significant as determined by *t* test

It is known that de novo FA synthesis in the plastids requires the supply of ATP and reducing power. Considering the fact that FA synthesis of *L. glauca* fruits specifically responded to different developing stages (Fig. 1c), we studied the time-course content patterns of ATP, ADP, NAD(P)H, and NAD(P) in developing fruits to determine a possible linkage between FA synthesis and provision of ATP and reducing power during development. We found that the contents of ATP and ADP were more sustained and continued to elevate toward the fully matured stage (Fig. 2a), but the contents of NADPH and NADP^+^ peaked at 125 DAF and then declined (Fig. 2b). Also, NADH and NAD^+^ levels first increased and then remained stable after 125 DAF (Fig. 2c). Interestingly, the ratios of NADPH/NADP^+^, NADH/NAD^+^, ATP/ADP, and ATP/NADPH were not statistically changed during fruit development (Fig. 2d), implying the maintenance of reduction–oxidation (redox) homeostasis in developing *L. glauca* fruits.

### Sequencing strategy and de novo assembly of developing fruits

To explore the molecular regulatory mechanism of FA biosynthesis and oil accumulation in developing *L. glauca* fruits for the development of woody biodiesel, we preformed transcriptome analysis by means of a combination of two different NGS platforms (454 and Illumina). Here, three fruit samples (50, 125, and 150 DAF) at critical periods of growth and oil accumulation (Fig. 1), and one mixed sample from seven different developments were selected for Illumina and 454 sequencing, respectively. By Illumina sequencing, 27,921,143 (average length of 92.85 bp), 29,036,556 (92.63 bp), and 24,297,910 (92.12 bp) clean short reads were, respectively, produced from three cDNA libraries (50, 125, and 150 DAF) after removing the low-quality reads and adaptor sequences (Table 2), all of which were together assembled using Trinity program, and 130,827 unigenes with mean length of 608.44 bp were obtained (Additional file 2: Table S2). In addition, by Venn diagram analysis, 102,258 unigenes were identified to be expressed in the whole developing stage, while 3488, 1056, and 843 unigenes were expressed specifically at 50, 125, and 150 DAF, respectively (Additional file 3: Figure S1). As for 454 sequencing, a total of 957,341 trimmed long reads (average length of 518.48 bp) were obtained from one cDNA library of mixed sample of seven different developing fruits (Table 2). By means of Newbler2.6, all the obtained high-quality long reads was assembled into 70,432 unigenes (15,729 unigenes >1000 bp) with mean length of 822.74 bp (Additional file 4: Table S3).

**Table 2:** Statistics of trimmed reads and unigenes of *L.glauca* fruits by different sequencing strategies

Sequencing strategy and assembly	Trimmed reads		Unigenes	
	Number	Mean length (bp)	Number	Mean length (bp)
Illumina sequencing				
50 DAF	27,921,143	92.85	130,827	608.44
125 DAF	29,036,556	92.63		
150 DAF	24,297,910	92.12		
454 sequencing	957,341	518.48	70,432	822.74
Short- and long-read assembly			60,031	1061.95

Three representative fruit samples from 50, 125 and 150 DAF and one mixed sample from seven different developing stages were selected for Illumina and 454 sequencing, respectively. The unigenes separately obtained from long read and short read (201,259 in total) was reconciled by TGICL software

To reconstruct the longer length of unigenes for accurate identification and functional analysis, all the above unigenes separately obtained from long read and short read (201,259 in total) were reconciled using TGICL software, resulting in 60,031 unigenes (22,916 unigenes >1000 bp) with average length of 1061.95 bp to define a minimal reference transcriptome for developing *L. glauca* fruits (Additional file 5: Table S4), indicating that the assembly strategy by a combination of 454 and Illumina sequencing provide more optimal results than each algorithm separately, which will contribute to reveal the complex regulatory mechanism of FA synthesis and oil accumulation in developing fruits of *L. glauca* for the development of woody biodiesel.

### Functional annotation and characterization of unigenes involved in oil synthesis of developing fruits

To better identify the unigenes to be involved specifically in oil biosynthesis of developing fruits, all the obtained long-sequence unigenes (60,031 >200 bp) were annotated using BLAST algorithm with an E-value <10^−5^ and protein identity >30% in the public databases, of which 34,854 (58.06%) and 25,606 (42.65%) unigenes showed significant similarities to the known proteins in non-redundant protein (NR) and Swiss-Prot protein databases, respectively (Additional file 6: Figure S2). Of all the obtained unigenes, 34,916 (58.16%) homologous unigenes were annotated in at least one database, but those unmatched unigenes (25,115, 41.84%) might be present as the putative specific novel genes for *L. glauca* or probably resulting from the shorter sequences with a lack of characterized protein domain to get BLAST hits [32, 39].

To classify the functions of all the annotated unigenes, we carried out the analysis of Gene Ontology (GO), Clusters of Orthologous Groups (COG), and Kyoto Encyclopedia of Genes and Genomes (KEGG). The resulting 22,851 (38.07%) unigenes were assigned into three main GO categories and 66 subcategories; 22,169 (36.93%) unigenes into 26 COG functional categories; and 8899 (14.81%) unigenes into 271 KEGG pathways, and 859 kinds of enzymes (Additional file 6: Figure S2). All these data emphasized the effectiveness of our sequencing strategy, assembly, and annotation processes. Importantly, we identified a total of 198 unigenes relevant to oil synthesis in developing fruits, mostly involved in photosynthesis, sucrose cleavage, glycolysis, OPPP, TCA cycle, acetyl-CoA production, metabolite transport, oxidative phosphorylation, FA biosynthesis, TAG assembly, and transcriptional regulation as well as the synthesis and utilization of ATP and reducing power, implying a complex transcript regulatory mechanism of carbon allocation and energy supply for oil synthesis in developing fruits of *L. glauca*.

### Carbon assimilation and sucrose cleavage for fruit development and FA synthesis

**Fig. 3:**
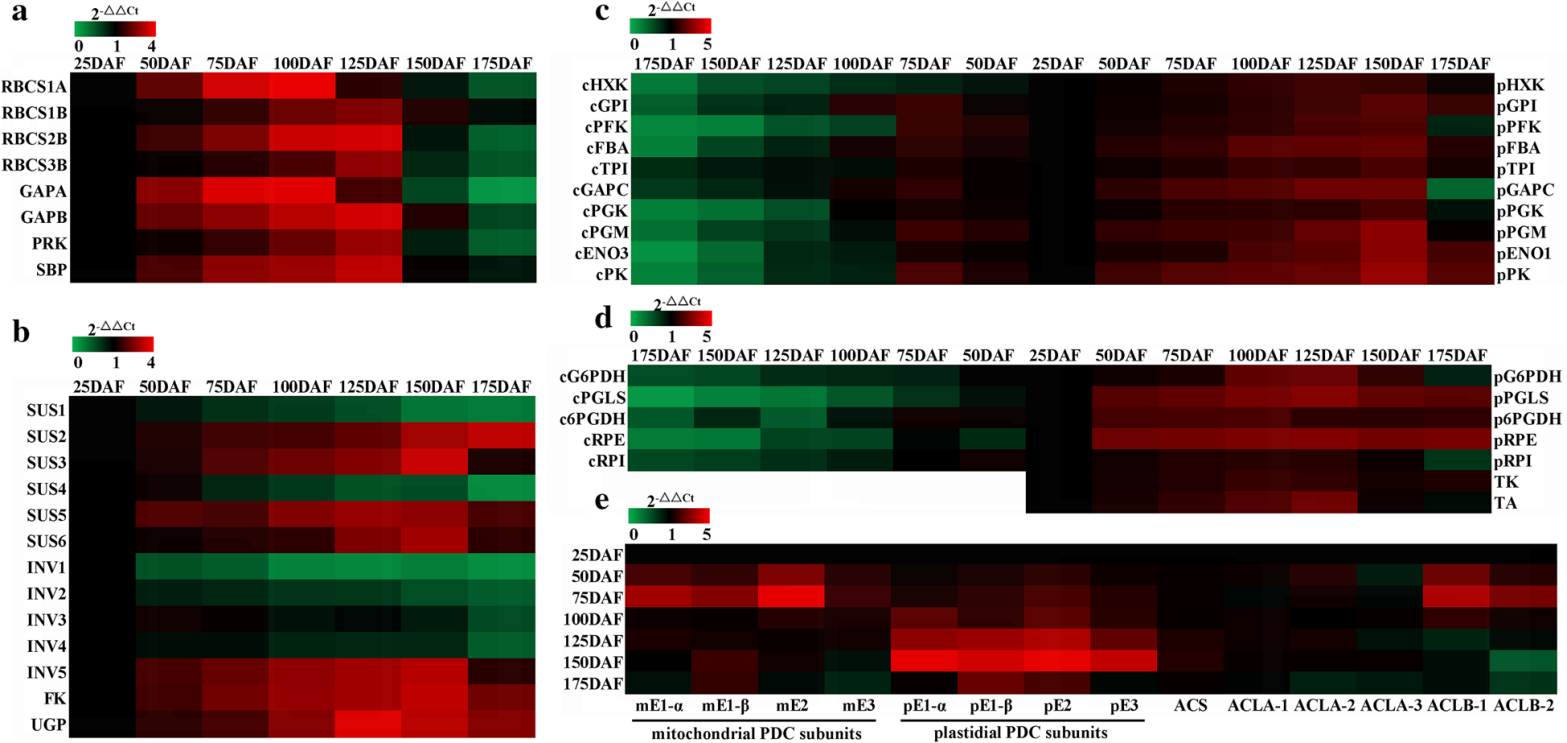
Transcriptional expression analysis for enzymes involved in carbon assimilation and partitioning in developing *L. glauca* fruits by qRT-PCR. **a** Differential expressions for genes involved in carbon assimilation. **b** Differential transcript patterns for enzymes related to sucrose cleavage. **c** Comparative analysis of transcript levels for enzymes in both cytosolic and plastidial glycolysis. **d** Comparative analysis of transcript levels for enzymes in both cytosolic and plastidial OPPP. **e** Differential transcript profiles for alternative enzymes involved in acetyl-CoA generation. The genes encoding for large subunit ribosomal protein L32e and ubiquitin-conjugating enzyme (UBC) were used as internal controls. The relative expression values in heatmap were counted as 2^−△△Ct^. The cytosolic (c), plastidial (p), or mitochondrial (m) isoforms of the enzymes are indicated by a prefix in **c**, **d,** or **e,** respectively

Sucrose, the major transport form of assimilated carbon in plants, is synthesized in photosynthetic tissues [15]. Here, a combination of transcriptome sequencing and temporal transcript analysis indicated that the genes for Rubisco small subunits (RBCS1A and RBCS1B/2B/3B), sedoheptulose-bisphosphatase (SBP), phosphoribulokinase (PRK), and GAPDH subunits (GAPA and GAPB) in Calvin cycle were highly expressed at 50–125 DAF, but declined during later stages (Fig. 3a; Additional file 7: Table S5), revealing a high capability of photosynthetic carbon assimilation specifically at early–middle development of fruits. In general, sucrose utilization is initiated from its cleavage by several isoforms of SUS and INV [17]. INV can be classified into two groups (acid and alkaline/neutral INV), and irreversibly hydrolyzes sucrose into glucose and fructose, while SUS (only in cytosol) catalyzes reversible conversion of sucrose and UDP to fructose and UDP-glucose (UDPG). Here, we identified six cytosolic SUS isoforms (SUS1-6) and 5 INV isoforms (cell-wall INV1/2/5 and vacuolar INV3/4) with differential transcripts in developing fruits, where most SUS isoforms (expect SUS1/4) displayed high transcript at 50–150 DAF, but only INV5 was highly expressed during the whole developing period (Fig. 3b; Additional file 8: Table S6). Also, fructokinase (FK) and UDPG pyrophosphorylase (UGP) (only present in cytosol) showed transcript abundance in developing fruits, whereas cytosolic hexokinase (HXK) was downregulated (Fig. 3b, c). Our results indicated that SUS, as the preferred sucrose-cleaving enzyme, may be responsible for feeding assimilated carbon from sucrose to hexoses (UDPG and Fru) in developing fruits of *L. glauca*.

### Transcript profiles for enzymes involved in PYR provision for FA synthesis in developing fruits

In plants, hexose phosphates generated from sucrose cleavage can be mainly metabolized via OPPP or glycolysis in both cytosol and plastid [13]. It is known that glycolysis implicates in a series of regulatory enzymes, such as ATP-dependent phosphofructokinase (PFK), fructose-bisphosphate aldolase (FBA), phosphoglycerate kinase (PGK), triosephosphate isomerase (TPI), GAP dehydrogenase (GAPC), enolase (ENO), and pyruvate kinase (PK), which may provide the large amounts of PYR required for high oil synthesis in oilseed plants [25, 27, 48], In this work, the genes coding for all known enzymes of a complete glycolytic pathway in both cytosol and plastid were characterized by a combination of 454 and Illumina sequencing analysis (Additional file 8: Table S6). Thus, it was important to determine the contribution of cytosolic or plastidial glycolysis to provide PYR for FA synthesis in developing fruits. We performed qRT-PCR to analyze the temporal transcript profiles of enzymes between two glycolytic pathways during fruit development, and found that the transcripts for plastidial HXK, GPI, PFK, FBA, TPI, GAPC, PGK, PGM, ENO1, and PK gradually increased with higher abundance at 75–150 DAF, but all cytosolic isoforms (except HXK) were upregulated specifically at 50–75 DAF (Fig. 3c). The present results suggested the differential transcriptional regulation between plastidial and cytosolic glycolysis in developing *L. glauca* fruits.

Also noteworthy was an involvement of OPPP in providing GAP for FA synthesis in plants [49–51]. Here, the genes for G6P dehydrogenase (G6PDH), 6-phosphogluconolactonase (PGLS), 6-phosphogluconate dehydrogenase (6PGDH), ribulose-5-phosphate epimerase (RPE), and ribose 5-phosphate isomerase (RPI) were identified in both cytosolic and plastidial OPPP of developing *L. glauca* fruits, while transaldolase (TA) and transketolase (TK) only presented in the plastids (Additional file 8: Table S6), revealing a complete plastidial OPPP in developing fruits. To explore whether OPPP in cytosol or plastid was involved specifically in providing carbon flux from hexose to GAP into FA biosynthetic pathway in developing fruits, their temporal transcript patterns were analyzed in different developing stages by qRT-PCR. It was observed that plastidial 6PGDH, PGLS, 6PGDH, RPE, and RPI showed higher transcript at 50–125 DAF, but most cytosolic isoforms were downregulated during the development (Fig. 3d), emphasizing that plastidial OPPP may be essential for FA synthesis in developing fruits of *L. glauca*.

### Transcript profiles for enzymes involved in acetyl-CoA generation for FA synthesis in developing fruits

It was suggested that the possible routes for acetyl-CoA synthesis in plants may be via four alternative enzymes of PDC, ACL, ACS, or CA [13, 28, 29]. By integrating 454 and Illumina sequencing analysis, we characterized the orthologs of plastidial and mitochondrial PDC subunits (E1-α, E1-β, E2, and E3), plastidial ACS, and cytosolic ACL isoforms (ACLA-1, ACLA-2, ACLA-3, ACLB-1, and ACLB-2) in developing fruits, but none of the genes was annotated for plastidial CA (Additional file 9: Table S7). Thus, there exists a multiple mechanism of generating acetyl-CoA for FA synthesis during fruit development. To address the potential contribution of PDC, ACS, or ACL to acetyl-CoA for FA synthesis in developing fruits, their temporal transcript patterns were conducted on different developing stages by qRT-PCR. It was shown that the transcripts for plastidial PDC gradually increased with a greater elevation at 50–150 DAF, while high transcript for mitochondrial PDC was detected only at 50–75 DAF (Fig. 3e), revealing that the difference of PDC transcripts between mitochondria and plastid may respond specifically to different developing stages of fruits. Also, we noticed cytosolic ACLB subunits (ACLB-1/-2) with transcript abundance at early developing stage, whereas low transcript was observed for both plastidial ACS and cytosolic ACLA subunits during development (Fig. 3e). Our results indicated that plastidial PDC and cytosolic ACLB subunits (ACLB-1/-2) may contribute a major role in the provision of acetyl-CoA for FA synthesis in developing fruits of *L. glauca*.

### Synthesis and utilization of ATP and reducing power for de novo FA synthesis in developing fruits

**Fig. 4:**
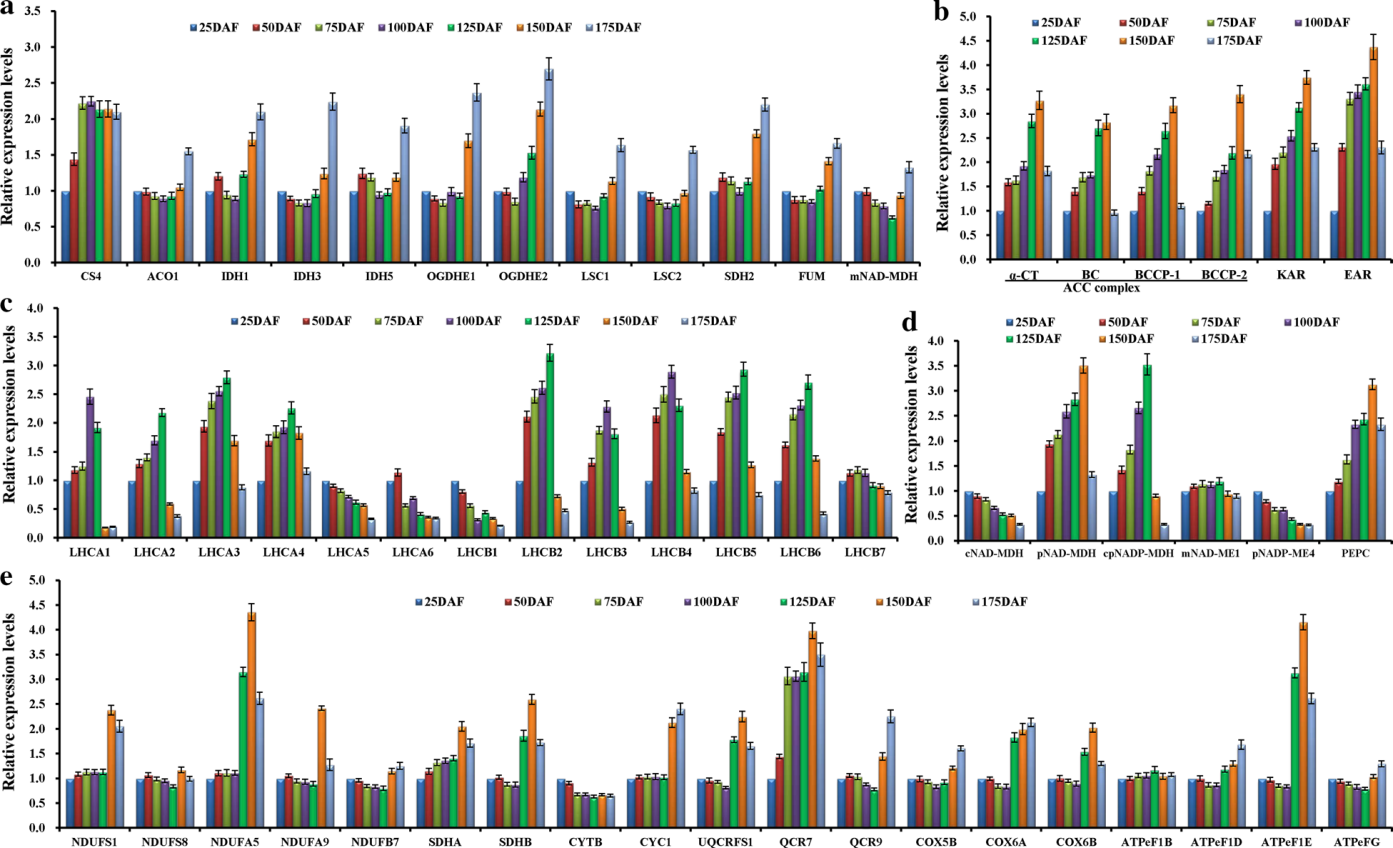
Transcriptional expression analysis of genes involved in energy mechanism in developing *L. glauca* fruits by qRT-PCR. **a** Temporal transcript patterns for enzymes in TCA cycle. **b** Temporal transcript patterns for enzymes involved in energy consumption during de novo FA synthesis. **c** Temporal transcript patterns of genes encoding for peripheral antenna proteins. **d** Temporal transcript changes for enzymes related to the Mal/OAA shuttle. **e** Temporal transcript patterns of genes involved in respiratory electron-transport chain. Both *L32e* and *UBC* genes were used as internal controls. The expression level from fruit sample at 25 DAF was arbitrarily set to 1.00 for standardization. *Error bars* are SDs of three technical replicates

In addition to carbon supply, de novo FA synthesis in plastids requires ATP, NADPH, and NADH [30]. To assess the potential sources of reducing power and ATP for de novo FA synthesis in developing *L. glauca* fruits, our analysis focused on differential temporal transcript patterns for genes that were related to energy provision during fruit development. Apart from plastidial glycolysis, PDC, and OPPP presented above (Fig. 3c–e), the genes for enzymes of mitochondrial TCA cycle were identified in developing fruits, including citrate synthase 4 (CS4), aconitate hydratase 1 (ACO1), isocitrate dehydrogenase (IDH1, IDH3, and IDH5), α-oxoglutarate dehydrogenase (OGDH) subunits (E1/2), succinyl-CoA synthetase (LSC1/2), succinate dehydrogenase (SDH2), fumarase (FUM), and NAD-dependent malate dehydrogenase (NAD-MDH) (Additional file 10: Table S8). Also, we found that *CS4* was highly expressed at 25–75 DAF, but the other enzymes increased transcripts after 125 DAF (Fig. 4a). Similarly, the components of respiratory electron-transport chain were significantly expressed at 150–175 DAF (Fig. 4e). Therefore, the contribution of mitochondrial oxidative phosphorylation to ATP production may be mainly at the late developing stage of *L. glauca* fruits.

Given the fact of developing *L. glauca* fruits with green at 25–125 DAF (Fig. 1a), it was needed to explore the role of photosynthesis during fruit development. We identified the genes of *LHCa 1*-*6* and *LHCb 1*-*7* encoding for six and seven chlorophyll *a/b*-binding proteins of light-harvesting complexes I and II (LHC I and II), respectively (Additional file 7: Table S5), of which most LHC genes (*LHCa1*-*4* and *LHCb2*-*6*) were highly expressed at 50–125 DAF (Fig. 4c). In addition, the genes involved in photosynthetic electronic transport and F-type ATPases as well as PSI and PSII complexes were annotated in developing fruits (Additional file 7: Table S5), and most of them were upregulated specifically at 50–125 DAF (Additional file 11: Figure S3). These results revealed the presence of linear electron flow in developing *L. glauca* fruits with the consequence of a production of NADPH and ATP.

Being surprised by the findings that the ratios of ATP/ADP, ATP/NADPH, NADPH/NADP^+^, and NADH/NAD^+^ were not significantly changed in developing fruits (Fig. 2d), we attempted to investigate whether or not the redox state was regulated by energy consumption during FA synthesis. Here, we analyzed the energetically expensive enzymes (ACC complex, KAR, and EAR), known in FA biosynthetic pathway. By functional annotation, the genes for ACC complex [biotin carboxylase subunit (BC), biotin carboxyl carrier protein (BCCP) isoforms (BCCP1, BCCP2), and carboxyltransferase subunits (α-CT and β-CT)], KAR and EAR were characterized in developing *L. glauca* fruits (Additional file 9: Table S7). Of note, the transcripts for ACC complex (BCCP1, α-CT, and BC), KAR, and EAR were upregulated specifically at 50–150 DAF (Fig. 4b), and exhibited a highly correlated temporal profile with the changes of ATP and reducing power contents (Fig. 2a–c), reflecting that the production and consumption of ATP and reducing power in developing *L. glauca* fruits were coordinated at the transcript level.

### Transporters involved specifically in carbon partitioning and energy metabolism in developing fruits

**Fig. 5:**
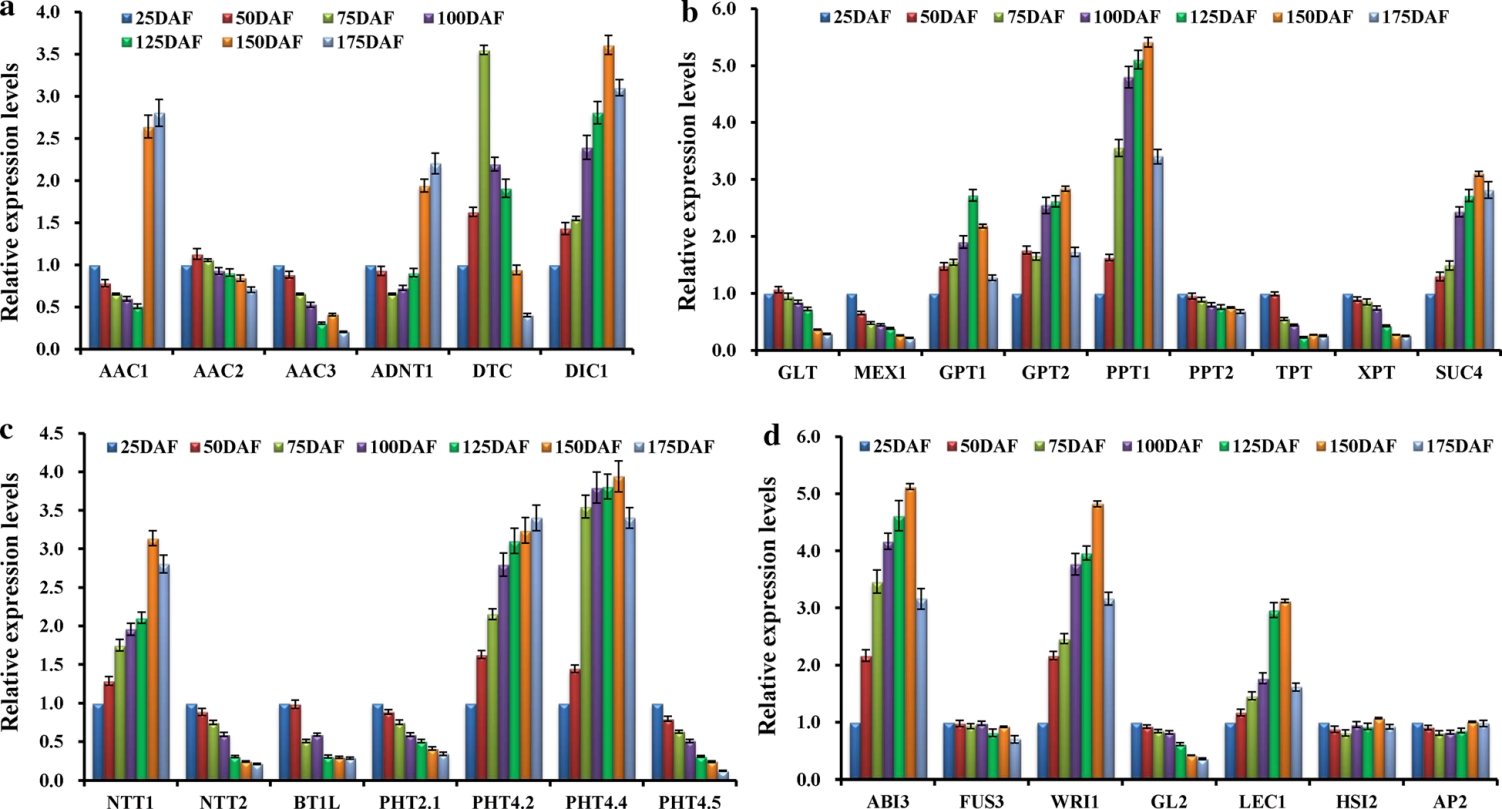
Transcriptional profiles for transporters and transcription factors in developing *L. glauca* fruits by qRT-PCR. **a** Temporal transcript profiles for mitochondrial metabolite transporters involved in TCA cycle, respiration, and ATP synthesis. **b** Temporal transcript profiles for plastidial transporters involved in interchange of metabolites between cytosol and plastid. **c** Temporal transcript profiles for plastidial transporters involved in the transports of adenine nucleotide and inorganic phosphate. **d** Temporal transcript profiles for transcription factors involved in oil accumulation. Both *L32e* and *UBC* genes were used as the internal controls, and the expression level from fruit sample at 25 DAF was arbitrarily set to 1.00 for standardization. *Error bars* are SDs of three technical replicates

In recent years, several mitochondrial transporters implicated in metabolite, respiration, and ATP synthesis have been identified in plants [52]. The genes encoding for orthologs of mitochondrial adenine nucleotide transporter (ADNT1), ADP/ATP carriers (AAC1, AAC2, and AAC3), dicarboxylate carrier (DIC), and dicarboxylate/tricarboxylate carrier (DTC) were marked here (Additional file 12: Table S9). Analysis of differential expression profiles showed that both AAC1 and ADNT1 transcripts were upregulated after 125 DAF, but DTC transcript increased before 75 DAF. Notably, DIC was detected with transcript abundance during the whole developing period (Fig. 5a). These results indicated that AAC1, ADNT1, DTC, and DIC may be considered as the important mitochondrial transporters for TCA cycle and ATP synthesis during fruit development of *L. glauca*.

It is important to note that in plants, the interchange of glycolytic intermediates between cytosol and plastid was involved in the selective transporters [13, 25–27], such as GLT, GPT, PPT, TPT, and XPT. Indeed, we identified some plastidial transporters in developing *L. glauca* fruits, including GLT, TPT, GPT (GPT1/2), PPT (PPT1/2), and XPT (Additional file 12: Table S9). Intriguingly, our qRT-PCR results revealed that GPT1, GPT2, and PPT1 were highly expressed at 50–150 DAF, but lower transcripts were detected for GLT, TPT, PPT2, and XPT during development (Fig. 5b), suggesting that GPT and PPT1 may contribute to provide glycolytic substrate hexose phosphate (G6P) and intermediate (PEP) from cytosol to plastid in developing fruits of *L. glauca*.

It was also noteworthy that plastidial transporters have played a role in the transport of adenine nucleotides or inorganic phosphates, such as nucleoside triphosphate transporters (NTT), brittle1-like transporter (BT1L), and phosphate (Pi) transporter (PHT) [53–58]. In this study, the differentially expressed genes encoding for BT1L, NTT isoforms (NTT1 and NTT2), one member of PHT2 family (PHT2.1), and three members of PHT4 family (PTH4.2, 4.4, and 4.5) were noted in developing *L. glauca* fruits (Additional file 12: Table S9; Fig. 5c). Of these, NTT1, PTH4.2, and PTH4.4 exhibited abundant transcripts at 50–150 DAF, but a strong decline was detected for BT1L, NTT2, PTH2.1, and PTH4.5 during fruit development. It could therefore be concluded that NTT1 and PTH4.2/4.4 may play a role in the transports of adenine nucleotide and Pi in developing fruits of *L. glauca*.

### Transcript profiles for enzymes and transcription factors involved in oil synthesis in developing fruits

**Fig. 6:**
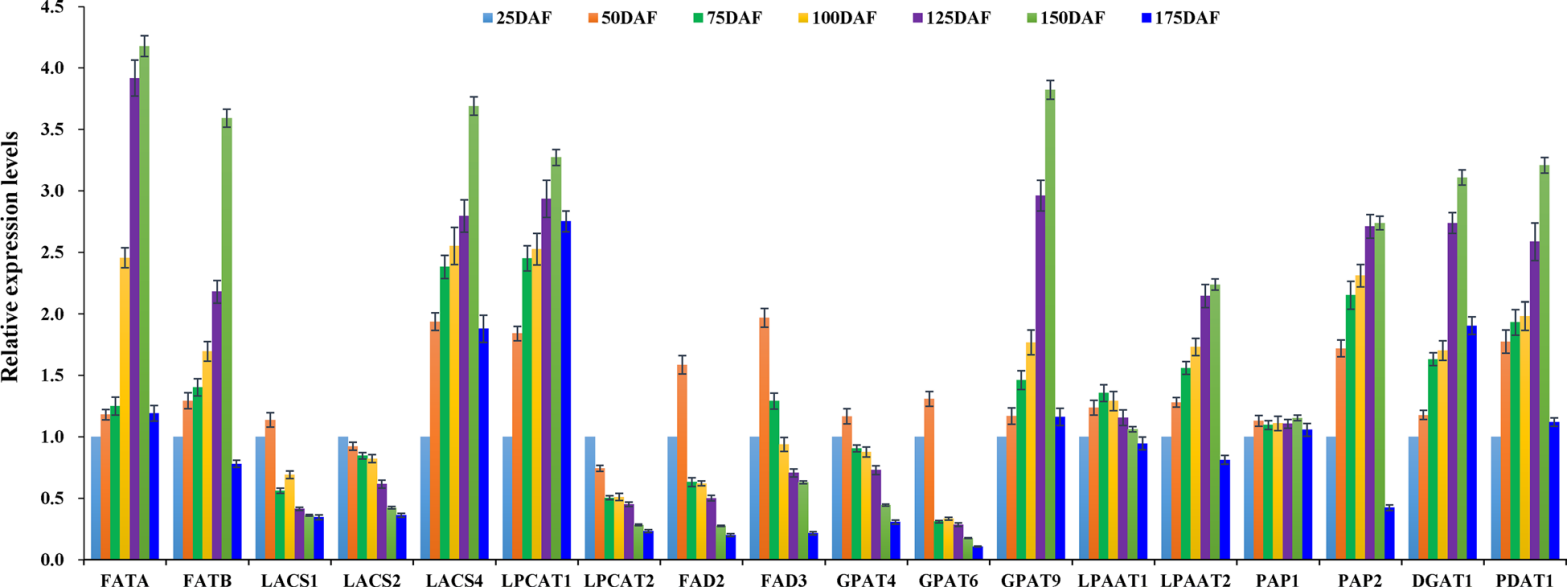
Transcriptional analysis for enzymes involved in TAG assembly in developing *L. glauca* fruits by qRT-PCR. Both *L32e* and *UBC* genes were used as the internal controls, and the expression level from fruit sample at 25 DAF was arbitrarily set to 1.00 for standardization. *Error bars* are SDs of three technical replicates

It is of interest to note that the synthesized free FAs by FATA/B thioesterase are exported from plastid to cytosol and then converted to the fatty acyl-CoAs by long-chain acyl-CoA synthetase (LACS), and destined to TAG assembly. In this study, the abundant transcript was characterized for FATA/B and LACS4 at 50–150 DAF (Additional file 13: Table S10; Fig. 6), revealing that FATA/B was vital to generate free FAs, and LACS4 may be as the major LACS isoform responsible for export of FAs from plastid to endoplasmic reticulum (ER). Also, high transcript of FA desaturase 2/3 (FAD2/3) for polyunsaturated FA synthesis were observed at 25–50 DAF (Fig. 6), paralleled to high content of polyunsaturated FAs (C18:2 and C18:3) in developing fruits (Fig. 1d), indicating its importance for polyunsaturated FA accumulation in early developing fruits. It is known that both G3P and the acyl chains are used for TAG assembly by a series of membrane-associated reactions in ER. We identified abundant transcript for G3P acyltransferase 9 (GPAT9), lysophosphatidyl acyltransferase 2 (LPAAT2), phosphatidate phosphatase 2 (PAP2), phospholipid:diacylglycerol acyltransferase 1 (PDAT1), and diacylglycerol acyltransferase 1 (DGAT1) at middle-late stage (75–150 DAF) (Fig. 6), implying the significant contribution of these to TAG assembly for high oil accumulation in developing *L. glauca* fruits.

Also of note was the involvement of transcription factors (TFs) in oil biosynthesis during fruit development of *L. glauca*. We analyzed all the unigenes by BLASTX against AGRIS database, and annotated TFs (ABI3, WRI1, LEC1, FUS3, HSI2, AP2, and GL2) in developing *L. glauca* fruits (Additional file 14: Table S11), which was known for the regulation of plant oil accumulation [16, 32]. Importantly, it was found that the transcripts of ABI3, LEC1, and WRI1 were upregulated at 50–150 DAF, whereas FUS3, HSI2, AP2, and GL2 all showed downregulation during fruit development (Fig. 5d). Thus, ABI3, LEC1, and WRI1 may play the important roles in regulating oil synthesis during fruit development of *L. glauca*.

## Discussion

In the current study, the developing fruits of *L. glauca* was used as a novel specific experimental material, and a subsequent analysis of oil content, FA composition and biodiesel properties during fruit development (Fig. 1; Table 1) revealed the oils from *L. glauca* fruits with a high quality and quantity as a novel potential source of woody biodiesel feedstock in China, which corresponded to the previous studies on traditional woody oil plants such as *P. chinensis*, *P. sibirica* and *J. curcas* [2–4, 32]. However, the complex regulatory mechanism of carbon flux and energy source required for oil synthesis in developing *L. glauca* fruits is poorly understood to date. Although NGS platforms have been recently used for transcriptional studies in several oilseed plants [16, 31, 32], long-read sequence data are needed for transcriptome assembly in non-model species for which a reference genome is not available [2–4, 32]. In this work, a combination of 454 and Illumina sequencing assembly strategies was performed to characterize a minimal reference transcriptome for developing *L. glauca* fruits. We obtained 60,031 unigenes with an average length of 1061.95 bp (Additional file 4: Table S3), which was not only longer than 608.44 and 822.74 bp from our Illumina and 454 sequencing, respectively (Table 2), but also than those reported in yellow horn (462 bp) [39] and Siberian apricot (651 bp) [42] by 454 sequencing. Our data indicated that the combined 454 and Illumina platforms could provide superior results to elevate coverage of *L. glauca* fruit transcriptome. Thus, the available assembly strategy and reliable function annotation could allow us to elucidate the molecular regulatory mechanism of carbon partitioning and energy supply for oil synthesis in developing *L. glauca* fruits.

### Sucrose cleavage specifically responded to the growth and oil synthesis of developing fruits

In most plants, carbon is supplied to the heterotrophic organs mostly as sucrose from photosynthetic tissues [15]. Our observations that high transcripts for chloroplastic RBCS, GAPDH, SBP, and PRK of Calvin cycle were identified between 25 and 125 DAF (Fig. 3a) and that their profiles were temporally correlated with the changes in growth tendency (Fig. 1b) and oil content of developing fruits (Fig. 1c) indicated that Calvin pathway was vital to provide carbon source (sucrose) for the development, growth, and oil synthesis at early–middle developing stage of fruits. It is known that the sucrose-cleaving enzymes are crucial for the development, growth, and carbon partitioning [15]. Given that most SUS isoforms (expect SUS1/4) showed high transcript during fruit development, while all INV isoforms (excerpt for INV 5) were downregulated (Fig. 3b), it seems certain that SUS rather than INV was the preferred enzyme responsible for initial sucrose cleavage in developing *L. glauca* fruits. Evidence for this conclusion was the finding that transcript level of cytosolic FK (upregulation) was higher than that of HXK (downregulation), and transcript abundance was marked for cytosolic UGP, an enzyme for conversion of UDPG to G1P (Fig. 3b, c). These outcomes, together with high expression for sucrose transporter SUC4 during fruit development (Fig. 5b), showed that the metabolism of imported sucrose via the highly coordinated transcripts of SUS, FK, and UGP could help to generate one cytosolic hexose phosphate pool for glycolysis in developing fruits of *L. glauca*, as was also noted in developing oilseeds of canola, Arabidopsis, *Ricinus communis*, *Euonymus alatus*, *Tropaeolum majus*, and *B. napus* by the EST analysis [31, 59–63]. Interestingly, a similar high transcript pattern was identified among four SUS isoforms (SUS2, 3, 5 and 6) in middle–late developing fruits of *L. glauca* (Fig. 3b), corresponding to the rapid phase of oil synthesis (Fig. 1d), emphasizing that they may specifically involve in the provision of carbon source (UDPG and Fru) for oil synthesis during fruit development. In addition, only cell–wall INV5 of our annotated five INV isoforms was detected with high transcript during fruit development (Fig. 3b), and showed a similar trend in fruit growth (Fig. 1b), implying that INV5 was mainly responsible for the growth and development of *L. glauca* fruits, as also noted in tomato fruits [17]. Together, the abundantly coordinated transcripts of SUS and INV isoforms (SUS2/3/5/6 and INV5) may specifically respond to carbohydrate availability for the growth, development, and FA synthesis in developing *L. glauca* fruits.

### Alternative carbon flux from hexose to PYR for FA synthesis in developing fruits

In recent years, studies on the utilization of metabolites for FA synthesis by plastids isolated from different plants have shown that the metabolites (G6P, PEP, PYR, acetate, or Mal) can be used as carbon source for high ratio of FA synthesis [13, 19–24]. However, up to now, reports about this situation in developing fruits are very scarce. Here, the observed low transcript of plastid ACS in developing *L. glauca* fruits (Fig. 3e) indicated that acetate was not a precursor for FA synthesis, as in the case for developing seeds of oilseed rape and sunflower [22–24]. Recently, three possible routes have been proposed for plastid PYR generation in developing oilseed rape seeds, including import of PYR from cytosol to plastid, carboxylation of Mal by plastid NADP-ME, or directly from plastid PEP by PK [64]. However, we were unable to annotate any genes for known plastid PYR transporter (Additional file 10: Table S8), revealing the lack of cytosolic PYR import in developing fruits of *L. glauca*. This coincided with the study in Arabidopsis developing seeds [62, 63], but contrasted sharply with developing *B. napus* seeds [23, 51, 64]. Also noteworthy was plastid NADP-ME4 with lower transcript during fruit development (Fig. 4d; Additional file 8: Table S6), suggesting its unimportance for plastid PYR production via carboxylation of Mal, as also reported in developing seeds of Arabidopsis and sunflower [14, 65–67].

Several evidences from recent work have indicated that a major flux through glycolysis in cytosol or plastid is expected to provide large amounts of PYR for high oil synthesis in oilseed plants [14, 16, 25, 27, 31, 32, 48], but it remains unclear to what extent both pathways are used in the conversion of hexose into oil biosynthetic pathway. In this work, we identified a higher number of transcripts of glycolytic enzymes in plastid than that in cytosol in developing fruits, and remarkably, plastidial PK transcript level showed threefold higher than that shown by cytosolic PK (Fig. 3c). This indicated that plastidial glycolysis may play a major role in providing PYR for FA synthesis, in which PYR was mainly derived from PEP by PK in developing *L. glauca* fruits, similar to earlier results in developing seeds of Arabidopsis, oilseed rape, and oil palm [14, 16, 30, 31, 51]. In support of this fact, the transcripts of both G6P transporters (GPT1/2) and PEP transporter (PPT1) increased over the active oil synthesis period in developing fruits (Figs. 1c, 5b), revealing that the imports of PEP and G6P from cytosol to plastid were critical for oil synthesis in developing *L. glauca* fruits. However, we found that PPT1 transcript level was on average twofold higher than that of GPT1/2 (Fig. 5b), pointing to a major carbon influx from cytosol into plastid at the level of PEP in developing *L. glauca* fruits, which was consistent with previous studies for developing Arabidopsis and oilseed rape seeds [14, 16, 49, 62, 63].

In addition to glycolysis, FA synthesis is also fed by an alternative sugar catabolic pathway, such as Rubisco shunt and OPPP [22, 30, 68, 69]. A completely active plastidial OPPP, reported in developing seeds of Arabidopsis and oilseed rape [14, 26, 30, 69], was identified here (Fig. 3d; Additional file 7: Table S5). This, integrated with high transcript for G6P transporter (GPT1/2) (Fig. 5b), implied that part of imported G6P from cytosol was partitioned into plastidial OPPP during fruit development. It was reported that during oil synthesis, a portion of imported G6P to plastid was mainly converted to precursor (GAP) of FA synthesis via the bypass of glycolysis by Rubisco [31, 49, 50, 67, 70]. The participation of such pathway in this work could be clearly demonstrated by higher number of transcripts for RBCS and PRK as well as other Calvin cycle enzymes (SBP and GAPA/B) during fruit development (Fig. 3a). The close correlation between their temporal profiles and the active oil synthesis period (Figs. 1c, 3a) indicated that the bypass Rubisco pathway of glycolysis and a complete active OPPP in plastids may be crucial for FA synthesis in developing *L. glauca* fruits.

Overall, transcript abundance for Rubisco, together with upregulation of both plastidial OPPP and glycolysis, could provide effective temporal carbon flux into oil biosynthetic pathway during fruit development of *L. glauca*.

### Acetyl-CoA formation specific for plastidial FA synthesis and cytosolic elongation in developing fruits

Acetyl-CoA, a key precursor for plastidial FA synthesis, cytosolic FA elongation, and mitochondrial TCA cycle [28], must be specifically synthesized in each subcellular compartment by alternative enzymes owing to membrane impermeability [29]. Thus, it is of particular importance to explore the mechanism of generating acetyl-CoA for FA synthesis in developing *L. glauca* fruits. Earlier studies have indicated that acetyl-CoA required for de novo FA synthesis in oilseed plants was mostly produced by plastid PDC [29, 31, 32, 51, 62, 63], which could be confirmed by our findings that plastidial PDC transcript was more abundant than mitochondrial isoforms (Fig. 3e), and exhibited a highly correlated temporal pattern with the actively FA synthesis in fruits at middle–late development (Fig. 1c, d). Given a higher proportion of hexose to PYR flux via plastidial glycolysis during fruit development (Fig. 3c), it could be seen clearly that the source of plastidial acetyl-CoA responsible for de novo FA synthesis in developing *L. glauca* fruits was mainly derived from glycolytic PYR via the action of PDC in plastids.

**Fig. 7:**
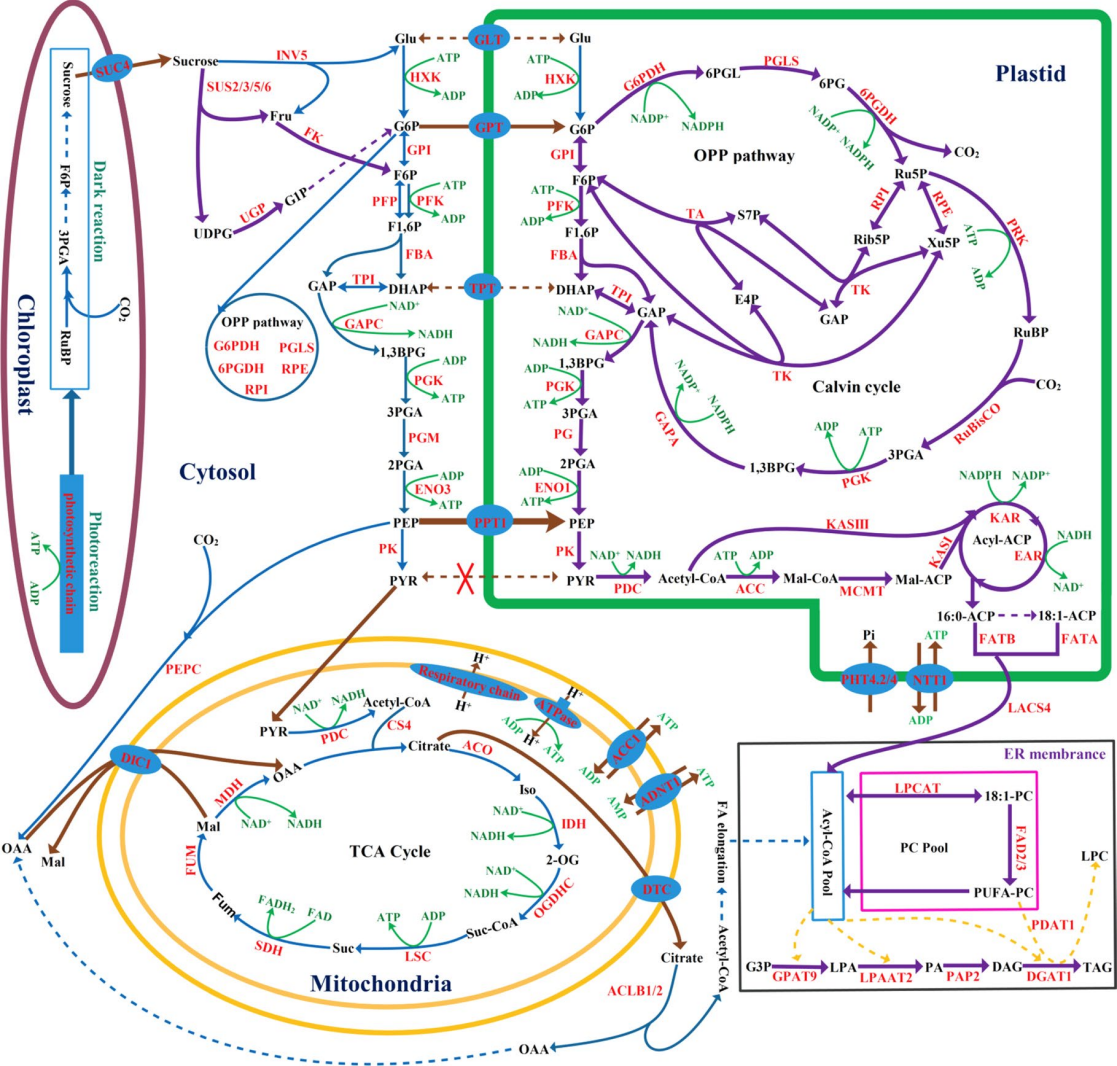
Characterization of central metabolic model in developing *L. glauca* fruits for the regulation of carbon partitioning and energy supply for oil synthesis. The identified metabolic routes of carbon flux allocation and energy provision for FA synthesis are based on the transcriptome data from Illumina and 454 sequencing, and temporal transcript pattern analysis by qRT-PCR. *Purple arrows* indicate carbon flux drains into oil synthesis, *brown arrows* represent the transports of metabolite and energy across intracellular membrane by specific transporters, and *green curved arrows* represent all reactions for generation and consumption of energy in FA biosynthetic pathway, oxidative pentose phosphate pathway (OPPP), glycolysis, and tricarboxylic acid (TCA) cycle. All transporters and enzymes involved in carbon flux allocation and energy provision for FA synthesis are shown in *red*. Abbreviations for the enzymes, metabolites, and transporters are as follows: *AAC* ATP/ADP carrier, *ACC* acetyl-CoA carboxylase, *ACLB* ATP-citrate lyase subunit B, *ACO* aconitate hydratase, *ADNT* adenine nucleotide carrier, *CS* citrate synthase, *DGAT* diacylglycerol (DAG) acyltransferase, *DIC* dicarboxylate carrier, *DTC* dicarboxylate/tricarboxylate carrier, *EAR* enoyl-ACP reductase, *ENO* enolase, *ER* endoplasmic reticulum, *FA* fatty acid, *FAD* FA desaturase, *FATA* fatty acyl-ACP thioesterase A, *FATB* fatty acyl-ACP thioesterase B, *FBA* fructose-bisphosphate (F1,6P) aldolase, *FK* fructokinase, *FUM* fumarase, *G6PDH* glucose-6-phosphate (G6P) dehydrogenase, *GAPA*/*C* glyceraldehyde 3-phosphate (GAP) dehydrogenase subunit A/C, *GLT* glycolipid transporter, *GPAT* G3P acyltransferase, *GPI* G6P isomerase, *GPT* G6P transporter, *HXK* hexokinase, *IDH* isocitrate dehydrogenase, *INV* invertase, *KAR* ketoacyl-ACP reductase, *LACS* long-chain acyl-CoA synthase, *LPAAT* lysoPA acyltransferases, *LPCAT* lysoPC acyltransferases, *LSC* succinyl-CoA synthetase, *MDH* malate (Mal) dehydrogenase, *ME* malic enzyme, *NTT* nucleoside triphosphate (NTP) transporter, *OGDHC* 2-oxoglutarate dehydrogenase, *6PGDH* 6-phosphogluconate (6PG) dehydrogenase, *PA* phosphatidic acid, *PAP* PA phosphohydrolases, *PC* phosphatidylcholine, *PDAT* DAG acyltransferase, *PDC* pyruvate (PYR) dehydrogenase complex, *PEPC* phosphoenolpyruvate (PEP) carboxylase, *PFK* ATP-dependent phosphofructokinase, *PFP* pyrophosphate phosphofructokinase, *PGK* phosphoglycerate kinase, *PGLS* 6-phosphogluconolactonase, *PGM* phosphoglycerate mutase, *PHT* phosphate (Pi) transporter, *PK* PYR kinase, *PPT* PEP transporter, *PRK* phosphoribulokinase, *RBCS* ribulose-1,5-bisphosphate carboxylase/oxygenase (Rubisco) small subunit, *RPE* ribulose-5-phosphate (Ru5P) epimerase, *RPI* ribose 5-phosphate isomerase, *SDH* succinate dehydrogenase, *SUC* sucrose transporter, *SUS* sucrose synthase, *TA* transaldolase, *TAG* triacylglycerol, *TK* transketolase, *TPI* triose phosphate isomerase, *TPT* triose phosphate transporters, *UGP* UDP-glucose (UDPG) pyrophosphorylase

The necessity of acetyl-CoA for the FA elongation in cytosol was mostly delivered from mitochondrial citrate, probably involved in citrate formation by CS in mitochondrial TCA cycle and export for cytosolic ACL cleavage by DTC transporter [14, 28, 51, 64, 65, 68]. This hypothesis could be supported by our observations that the coordinately upregulated transcripts for mitochondrial CS4 and DTC (Figs. 4a, 5a) and cytosolic ACLB (Fig. 3e) in early developing fruits was positively correlated with the synthesis of C20:4 FA (Fig. 1d). Also, mitochondrial dicarboxylate carrier DIC1, as Mal/OAA shuttle for import of cytosolic OAA destined to citrate synthesis [71], was detected with transcript abundance (Fig. 5a). Therefore, these results at transcript level revealed that in early-stage developing fruits of *L. glauca*, citrate was produced by a citrate shunt cycle in mitochondria and then imported into cytosol for ACLB cleavage to produce cytosolic acetyl-CoA pool for the synthesis of C20:4 FA (Fig. 7).

Taken together, strongly increased plastid carbon supply via glycolysis, in conjunction with the effective attribution of plastidial OPPP and cytosolic ACLB, was likely crucial for oil synthesis in developing *L. glauca* fruits.

### ATP and reducing power specifically for FA synthesis and energy homeostasis in developing fruits

The de novo synthesis of FAs in the plastid of plants generally requires stoichiometric amounts of ATP, NADPH, and NADH [13]. Current knowledge of a linkage between NADPH supply and FA synthesis by OPPP is mainly derived from G6P metabolism in the isolated plastids from oilseed rape and sunflower seeds [22, 30, 68, 69, 72, 73]. Our identification of a completely active plastidial OPPP at 50–125 DAF (Fig. 3d), the period was important for fruit FA synthesis (Fig. 1c), implying that most of NADPH supply for FA synthesis by plastidial OPPP was mainly at early–middle development. However, it was reported that plastidial OPPP could provide at most 9% of NADPH required for FA synthesis in oilseed rape seeds [14], suggesting that not all of NADPH for FA synthesis may be derived from OPPP. Also noteworthy was plastidial NADP-ME as the source of NADPH in developing *B. napus* seeds [24]. Unfortunately, we identified low transcript for plastidial NADP-ME4 (Fig. 4e), indicating that it may make negligible contribution to NADPH for FA synthesis in developing *L. glauca* fruits, as was noted in developing seeds of sunflower, Arabidopsis, and *B. napus* [14, 65–67]. Considering that developing fruits were green at 25–125 DAF (Fig. 1a), and high transcript for photosynthetic light reactions between 50 and 125 DAF (Additional file 7: Figure S3) was closely correlated to the oil synthesis period (50–150 DAF) (Fig. 1c), it was apparent that at early–middle development, *L. glauca* fruits had the capacity of photosynthesis to produce NADPH and ATP for oil synthesis, which coincided with previous results from of plant oilseeds by the microarray and EST analysis [50, 62, 63, 70]. However, transcript of photosystem was found to be correlated temporally with that of dark reaction rather than with those of energetically expensive enzymes (ACC, KAR, and EAR) (Figs. 3a, 4b). Combined with the report of carbon supply for fruit mostly as sucrose [16, 74], it could be interpreted to suggest that the chloroplasts of developing *L. glauca* fruits mainly provide carbon source for oil synthesis but probably make the relatively limited contribution to ATP and NADPH.

It is also intriguing to note that the sources of ATP and NADH can generate in plastids during the synthesis of acetyl-CoA from G6P [20, 22, 30], and ATP production via plastidial glycolysis was insufficient for plant FA synthesis [13, 20, 21, 75, 76]. Here, the transcripts for plastidial PDC and glycolysis-associated enzymes (GAPC, PGK, and PK) relevant to the productions of NADH and ATP increased strongly at 50–150 DAF (Fig. 3c, e), and importantly, their temporal patterns were positively correlated with fruit FA synthesis (Fig. 1c), indicating that plastidial glycolysis together with PDC were very like to provide most of ATP and NADH for de novo FA synthesis during fruit development of *L. glauca*. However, we also observed that the enzymes (IDH, OGDH, SDH2, and NAD-MDH) for NADH- and FADH_2_-producing reactions in TCA cycle showed a similar transcript pattern with the respiratory chain after 125 DAF (Fig. 4a, e). This finding, combined with developing fruits with dark brown (Fig. 1a) and the maximum synthesis of fruit FAs at late development (Fig. 1c), allowed us to speculate that mitochondria may supply a part of ATP for further FA synthesis in the absence of photosynthesis after 125 DAF. Also consistent with this conclusion, the transporters of ADNT1 and AAC1 involved in the export of mitochondrial ATP [77], were observed with transcript abundance at 150–175 DAF (Fig. 5a).

Maintenance of redox homeostasis plays a pivotal role for many metabolic processes in plants [78–81]. Our observations on the ratios of ATP/ADP, ATP/NADPH, NADPH/NADP^+^, and NADH/NAD^+^ with no significant change during fruit development (Fig. 2d), revealed the energy homeostasis in developing *L. glauca* fruits. Since plant membranes are broadly impermeable to NAD(P) and NAD(P)H, they can be indirectly transported through the reversible interconversion of Mal and OAA catalyzed by MDH [78–80]. Of our annotated four MDH isoforms (Additional file 9: Table S7), only plastid-localized NAD-MDH was highly expressed and significantly correlated to plastidial glycolysis and FA synthesis during fruit development (Figs. 1c, 3c and 4d), indicating that plastidial NAD-MDH was crucial for energy homeostasis during fruit FA synthesis, as could be evidenced by mutant studies of plastidial *NAD*-*MDH* for developing Arabidopsis seeds [78, 81].

### Coordinated transcripts of specific enzymes and transcription factors for oil accumulation in developing fruits

Although the biochemical pathway of FA synthesis and oil accumulation in plants has been well identified [13, 82], the molecular regulatory mechanism of high oil accumulation of plants is incompletely understood. In this study, the similar transcript patterns were identified for enzymes of sucrose cleavage, plastidial glycolysis, acetyl-CoA generation and de novo FA synthesis (Figs. 3, 4b), and these patterns coincided with TAG assembly-related genes (FATA/B, LACS4, GPAT9, LPAAT2, PAP2, PDAT1, and DGAT1) (Fig. 6) during fruit development. This indicated that allocation of carbon flux into FA synthesis destined to oil accumulation in developing *L. glauca* fruits were coordinately regulated at the transcript level. Indeed, the transcription factors (TFs) related to oil accumulation has been extensively studied [16, 32, 83–86]. Recently, transcriptomic analysis has shown that ABI3, WRI1, and LEC2 participated in positive regulation of the genes for oil synthesis in developing Siberian apricot seeds, while AP2 and GL2 acted as negative regulatory factors [32], as was the case for our experiments. We characterized the genes encoding for 7 TFs (ABI3, WRI1, LEC1, FUS3, HSI2, AP2, and GL2) with differential transcript profiles (Fig. 5d), of which only ABI3, LEC1, and WRI1 increased transcript throughout the time course of oil synthesis during fruit development of *L. glauca* (Figs. 1c, 5d), and thus were candidates for positive effectors of oil synthesis. It is important to note that WRI1, an AP2-domain-containing transcription factor, is known to be upregulated by LEC, LEC2, ABI3, and FUS3 in Arabidopsis [83, 87, 88]. In the present work, a high similar transcript pattern was marked among ABI3, LEC1, and WRI1 during fruit development (Fig. 5d), implying that the WRI1 gene might be a direct target of LEC1 or ABI3 in developing *L. glauca* fruits. This was apparently in contradiction with oil palm that WRI1 was likely to control oil synthesis independently of the upstream factors [16]. In addition, we found that most of enzymes for carbon flux allocation (sucrose cleavage, plastidial glycolysis, and acetyl-CoA synthesis), de novo FA synthesis, and TAG assembly exhibited a coordinated transcript profile with WRI1 during fruit development (Figs. 3, 4b, 5c and 6), suggesting that these genes may act as the targets of WRI1, similar to the results from other oilseed plants [16, 32, 83–86]. Taken together, WRI1 may play a major role in regulatory network of FA synthesis and oil accumulation in developing *L. glauca* fruits.

## Conclusions

In the present work, a concurrent analysis of temporal patterns for the growth, oil accumulation, and biodiesel fuel properties as well as redox homeostasis was conducted during *L. glauca* fruit development, which could provide valuable reference for exploring the regulatory mechanism of FA biosynthesis and oil accumulation in developing fruits for the development of woody biodiesel. Importantly, comprehensive characterization of transcriptome in developing *L. glauca* fruits was performed using a combination of two completely different NGS platforms, 454 and Illumina. De novo assembly of transcriptome provided 60,031 unigenes with average length of 1061.95bp, of which 198 expressed genes were relevant for carbon partitioning and energy provision for oil biosynthesis in developing *L. glauca* fruits. In addition, the application of an integrated transcriptome sequencing analysis and qRT-PCR detection has led to the identification of potential pathways, metabolite transporters, enzymes, and transcription factors in intermediary metabolism for oil biosynthesis, involved specifically in carbon flux allocation and redox homeostasis balance during the FA biosynthesis of developing *L. glauca* fruits. Together, our findings and metabolic model (Fig. 7) will be both a rich source of data and of considerable interest to those studying the molecular regulatory mechanism of oil accumulation in woody biodiesel plants.

## Methods

### Collection of plant materials

*Lindera glauca* fruits from different developing stages were collected from plus tree (LG-DZ02) located at DongZhai National Natural Reserve (E114°18′, N31°56′) of Henan Province, China. Flowers with the same anthesis were marked, and fruits were harvested at 25 (immature stage), 50, 75, 100, 125, 150, and 175 DAF (fully matured stage), respectively. The samples were immediately frozen in liquid nitrogen and stored at −80 °C until use.

### Determination of oil content and evaluation of biodiesel properties

About 50 g of fresh fruits from each sample (about 15 samples per accession) was used for oil extraction, and oil content and FA composition were determined using a previously described method [12]. The fruit weight was measured using an electronic balance, and fruit volume was determined with displacement method of drainage. Also, the biodiesel properties (IV, CN, CFPP, OS, and triangular prediction model) of fruit oils from different developing stages were evaluated as per our previously described method [47]. All the determinations were performed in triplicate.

### Assays of ATP, ADP, NAD(H), and NADP(H)

ADP and ATP were extracted from fruits by the trichloroacetic acid method, and the levels of ATP and ADP were measured as described previously [89]. The reduced pyridine nucleotides (NADPH and NADH) and the oxidized pyridine nucleotides (NADP^+^ and NAD^+^) were extracted by grinding 100 mg fresh weight of fruits with 1 ml of 0.1 M NaOH and 0.1 M HCl, respectively, and the contents were determined by the enzymatic cycling method of Matsumura and Miyachi [90]. All the determinations were performed in triplicate.

### cDNA library preparation and transcriptome sequencing and assembly

According to the detected results of oil contents, FA compositions, and biodiesel properties of developing fruits, the fruits from three representative periods (50, 125, and 150 DAF) were selected as experimental materials for Illumina sequencing. The equal weights of 3–5 biological fruit samples from every developing stage were mixed, and then total RNA was extracted using RNeasy Plant Mini Kits (Qiagen, Inc., USA). The obtained RNA was qualified and quantified using Nanodrop ND-1000 Spectrophotometer (N Wilmington, DE, USA), and all the samples showed a 260/280 nm ratio from 1.9 to 2.1. As for 454 sequencing, one mixed fruit sample from seven developing stages was used, and equal amounts of total RNA from every developing stage were mixed together for cDNA preparation. The construction and normalization of cDNA library (two independent biological replicates) were performed as previously described in our studies [32, 46]. Illumina and 454 sequencing were conducted on Illumina sequencing platform (HiSeq™ 2000) and 454 GS FLX Titanium genomic sequencer (Roche, Indianapolis, IN, USA), respectively. These data have been deposited in NCBI/SRA database under accession numbers: SRR5192952, SRR5179605, SRR5179607, and SRR2017832.

The raw reads from Illumina sequencing were filtered to obtain processed reads by removing the adapter sequences, low-quality sequences (reads with ambiguous bases ‘N’), and reads with more than 10% Q <20 bases. The 454 GS-FLX generated raw data were preprocessed to eliminate the relatively short sequences (<45 bp) and low-quality regions using the Newbler (http://454.com/products/analysis-software/index.asp) and Lucy (http://lucy.sourceforge.et/) software programs, and SeqClean (http://compbio.dfci.harvard.edu/tgi/software) was applied to trim the adapters and SMART primers used for reverse transcription. The clean reads from 454 and Illumina sequencing were assembled into unigenes using the Newbler and Trinity program, respectively. Finally, all the obtained unigenes by the two sequencing technologies were reconciled using TGICL software, and a minimal reference transcriptome for developing *L. glauca* fruits was defined.

### Functional annotation and reconciled algorithm of unigenes in developing fruits

The fruit unigenes were annotated using BLASTX alignment with an E-value cutoff of 10^−5^ against the known protein databases of NR, SWISS-PROT, TREMBL, AP, CDD, PFAM, and COG. Also, GO functional classifications were analyzed by GO terms (http://www.geneontology.org) using Blast2Go software, and KEGG pathway assignments were performed using BLAST all against Kyoto Encyclopedia of Genes and Genomes database. To obtain the longer length of unigenes for accurate identification and functional analysis of genes associated with carbon flux allocation and energy source for FA biosynthesis in developing fruits, all the relevant unigenes (from long- and short-read assemblies) involved in FA synthesis were reconciled by Sequencher software.

### Differential expression analysis of unigenes

The expression levels of unigenes were calculated using RPKMs (reads per kilobase transcriptome per million mapped reads) for eliminating the influences of gene length and sequencing level on the calculation of gene expression. The levels of unigenes expressions in the different samples were compared using the DESeq method described in our previous study [46].

### qRT-PCR assay

Total RNA was extracted as per the description for cDNA library preparation and was reverse transcribed using the Reverse transcription System (Promega). The amplified primers (Additional file 15: Table S12) were designed using PrimerQuest (http://www.idtdna.com/PrimerQuest/Home/Index) software at melting temperatures of 62 °C, and the absence of secondary structures was verified by the UNAFold program (http://eu.idtdna.com/UNAFold). The genes coding for large subunit ribosomal protein L32e and ubiquitin-conjugating enzyme (UBC) were used as inner references as described previously [46]. The qRT-PCR was conducted on 7500 Real-Time PCR System using SYBR Premix Ex Taq Kit (TaKaRa). Negative controls consisting of nuclease-free water instead of template, and reverse transcriptase controls prepared by substituting reverse transcriptase for nuclease-free water in cDNA synthesis step were included in all analyses for each primer pair. Three technical repetitions were performed for qRT-PCR.

## Additional files

**Additional file 1: Table S1.** Evaluation of the biodiesel fuel properties of oils from developing *L. glauca* fruits.

**Additional file 2: Table S2.** All unigenes obtained from 3 representative *L. glauca* fruit samples by Illumina sequencing.

**Additional file 3: Figure S1.** Number of differential expression genes of developing *L. glauca* fruits by Illumina sequencing.

**Additional file 4: Table S3.** All unigenes obtained from one mixed fruit sample of 7 developmental periods by 454 sequencing.

**Additional file 5: Table S4.** Unigenes obtained from developing *L. glauca* fruits by integrated 454 and Illumina sequencing analysis. The results indicated that the combined 454 and Illumina platforms could provide superior results than each NGS platform by separate to elevate coverage of the *L. glauca* fruit transcriptome.

**Additional file 6: Figure S2.** Length distribution and functional annotation of unigenes obtained from developing *L. glauca* fruits. (A) Comparative analysis of length distribution of unigenes generated from the two completely different sequencing strategies (Illumina and 454 sequencing) and assembly. The unigenes separately obtained from long read and short read (201,259 in total) was reconciled by TGICL software, which provided a catalogue of 60,031 unigenes with average length of 1061.95 bp to define a minimal reference transcriptome for developing *L. glauca* fruits. (B) Functional annotation of unigenes from BLAST searches against public databases. (C) Histogram presentation of Clusters of Orthologous Groups (COG) classification and a total of 22,169 unigenes were assigned to 26 classifications. (D) Histogram presentation of Gene Ontology (GO) classification, including 3 categories of biological process, cellular component, and molecular function.

**Additional file 7: Table S5.** Annotated information of genes involved in photosynthesis of developing *L. glauca* fruits. These genes mainly include light-harvesting complex I and II (LHC I and II), PSI and PSII complex, photosynthetic electronic transport, F-type ATPase and dark reaction.

**Additional file 8: Table S6.** Annotated information of genes for enzymes for carbon partitioning in developing *L. glauca* fruits. These genes include sucrose cleavage, glycolysis and oxidative pentose phosphate pathway (OPPP).

**Additional file 9: Table S7.** Annotated information of genes encoded for other enzymes in developing *L. glauca* fruits. These genes mainly involved in the acetyl-CoA formation, Mal/OAA shuttle and energy consumption for FA synthesis.

**Additional file 10: Table S8.** Annotated information of genes involved in mitochondrial metabolism of developing *L. glauca* fruits. These genes include TCA cycle and respiratory electron-transport chain.

**Additional file 11: Figure S3.** Transcript patterns of genes involved in photosynthetic light reaction in developing *L. glauca* fruits.

**Additional file 12: Table S9.** Annotated information of genes encoded for the transporters in developing *L. glauca* fruits. They involved specifically in carbon partitioning and energy metabolism.

**Additional file 13: Table S10.** Annotated information of genes involved in TAG assembly in developing *L. glauca* fruits. They involved specifically in the reactions for the generation of long-chain acyl-CoA, polyunsaturated FA, phosphatidic acid (PA), diacylglycerol (DAG) and triacylglycerol (TAG).

**Additional file 14: Table S11.** Annotated information of transcription factors involved in oil synthesis during fruit development.

**Additional file 15: Table S12.** The information of all primers used in this study for qRT-PCR analysis.

## Abbreviations

AAC: ATP/ADP carrier
ACC: acetyl-CoA carboxylase
ACLB: ATP-citrate lyase subunit B
ACO: aconitate hydratase
ADNT: adenine nucleotide carrier
CS: citrate synthase
DGAT: diacylglycerol (DAG) acyltransferase
DIC: dicarboxylate carrier
DTC: dicarboxylate/tricarboxylate carrier
EAR: enoyl-ACP reductase
ENO: enolase
ER: endoplasmic reticulum
FA: fatty acid
FAD: FA desaturase
FATA: fatty acyl-ACP thioesterase A
FATB: fatty acyl-ACP thioesterase B
FBA: fructose-bisphosphate (F1,6P) aldolase
FK: fructokinase
FUM: fumarase
G6PDH: glucose-6-phosphate (G6P) dehydrogenase
GAPA/C: glyceraldehyde 3-phosphate (GAP) dehydrogenase subunit A/C
GLT: glycolipid transporter
GPAT: G3P acyltransferase
GPI: G6P isomerase
GPT: G6P transporter
HXK: hexokinase
IDH: isocitrate dehydrogenase
INV: invertase
KAR: ketoacyl-ACP reductase
LACS: long-chain acyl-CoA synthase
LPAAT: lysoPA acyltransferases
LPCAT: lysoPC acyltransferases
LSC: succinyl-CoA synthetase
MDH: malate (Mal) dehydrogenase
ME: malic enzyme
NTT: nucleoside triphosphate (NTP) transporter
OGDHC: 2-oxoglutarate dehydrogenase
6PGDH: 6-phosphogluconate (6PG) dehydrogenase
PA: phosphatidic acid
PAP: PA phosphohydrolases
PC: phosphatidylcholine
PDAT: DAG acyltransferase
PDC: pyruvate (PYR) dehydrogenase complex
PEPC: phosphoenolpyruvate (PEP) carboxylase
PFK: ATP-dependent phosphofructokinase
PFP: pyrophosphate phosphofructokinase
PGK: phosphoglycerate kinase
PGLS: 6-phosphogluconolactonase
PGM: phosphoglycerate mutase
PHT: phosphate (Pi) transporter
PK: PYR kinase
PPT: PEP transporter
PRK: phosphoribulokinase
RBCS: ribulose-1,5-bisphosphate carboxylase/oxygenase (Rubisco) small subunit
RPE: ribulose-5-phosphate (Ru5P) epimerase
RPI: ribose 5-phosphate isomerase
SDH: succinate dehydrogenase
SUC: sucrose transporter
SUS: sucrose synthase
TA: transaldolase
TAG: triacylglycerol
TK: transketolase
TPI: triose phosphate isomerase
TPT: triose phosphate transporters
UGP: UDP-glucose (UDPG) pyrophosphorylase
