# Supplementary figures and images for: Integrated analysis of 454 and Illumina transcriptomic sequencing characterizes carbon flux and energy source for fatty acid synthesis in developing Lindera glauca fruits for woody biodiesel
Source: Biotechnol Biofuels. 2017 May 25;10:134. doi: 10.1186/s13068-017-0820-2 (PMC5445305; doi:10.1186/s13068-017-0820-2)

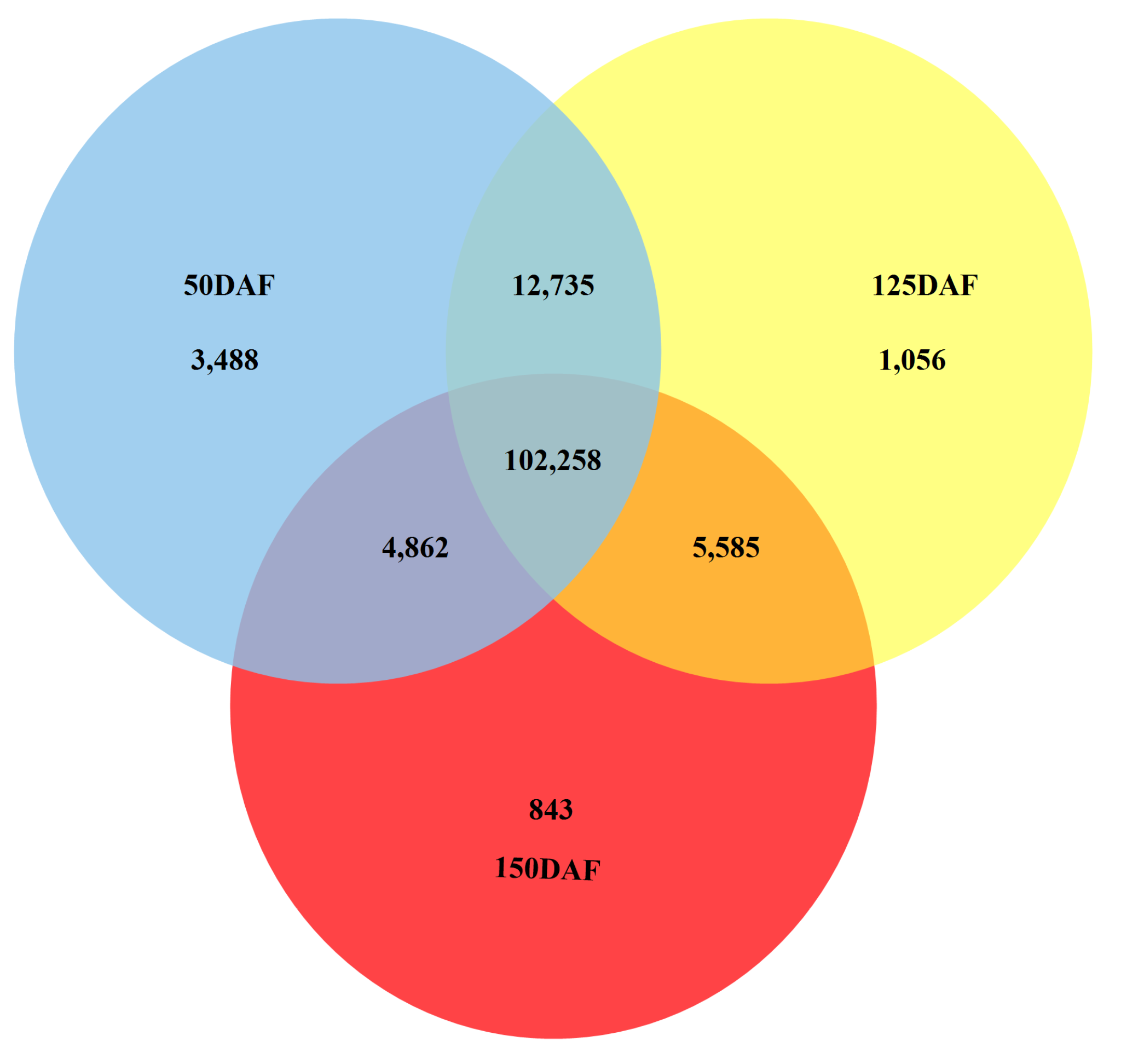

Supplement: Supplementary file 3 — Additional file 3: Figure S1. Number of differential expression genes of developing L. glauca fruits by Illumina sequencing. [file 13068_2017_820_MOESM3_ESM.tiff]

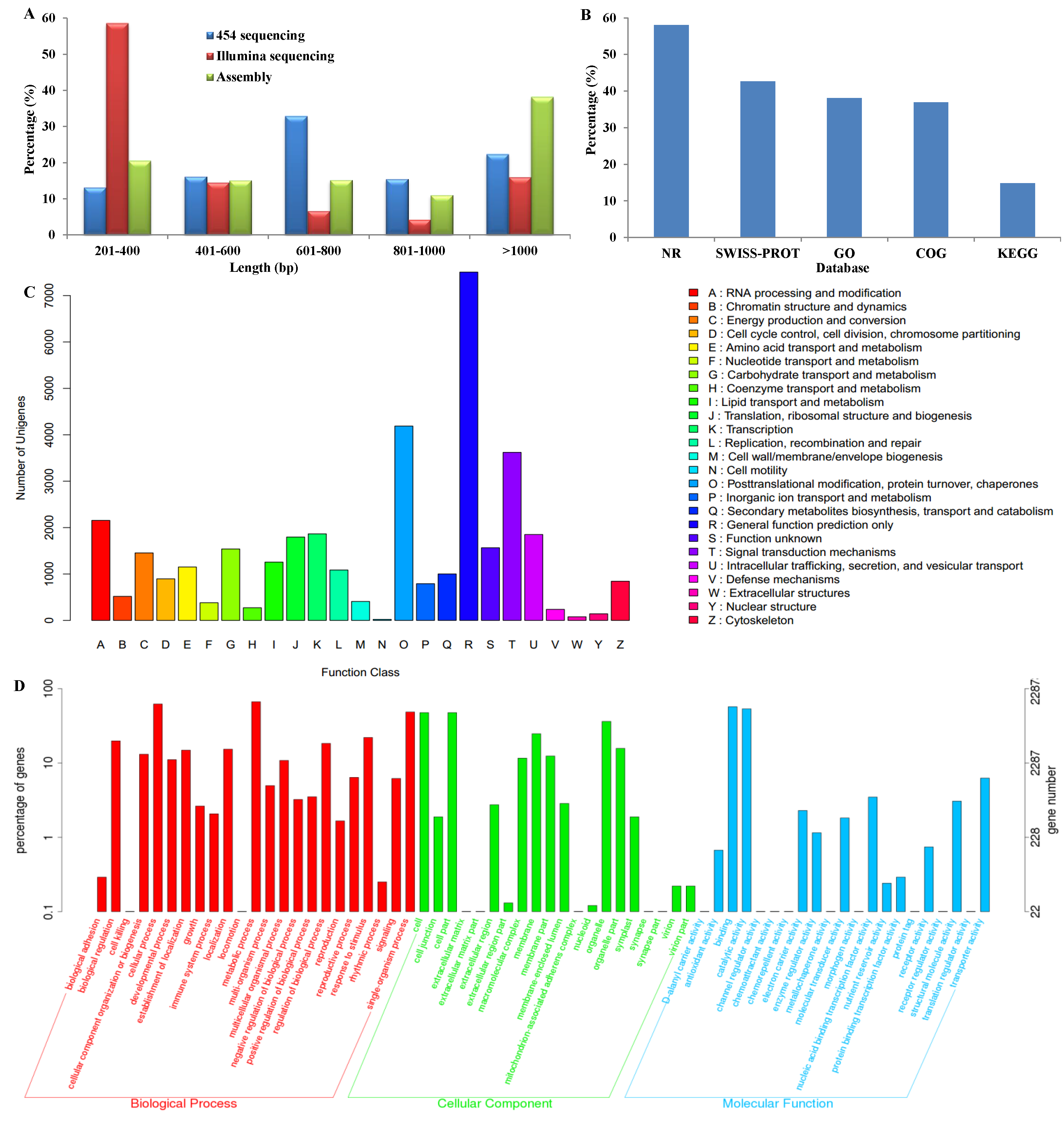

Supplement: Supplementary file 6 — Additional file 6: Figure S2. Length distribution and functional annotation of unigenes obtained from developing L. glauca fruits. (A) Comparative analysis of length distribution of unigenes generated from the two completely different sequencing strategies (Illumina and 454 sequencing) and assembly. The unigenes separately obtained from long read and short read (201,259 in total) was reconciled by TGICL software, which provided a catalogue of 60,031 unigenes with average length of 1061.95 bp to define a minimal reference transcriptome for developing L. glauca fruits. (B) Functional annotation of unigenes from BLAST searches against public databases. (C) Histogram presentation of Clusters of Orthologous Groups (COG) classification and a total of 22,169 unigenes were assigned to 26 classifications. (D) Histogram presentation of Gene Ontology (GO) classification, including 3 categories of biological process, cellular component, and molecular function. [file 13068_2017_820_MOESM6_ESM.tiff]

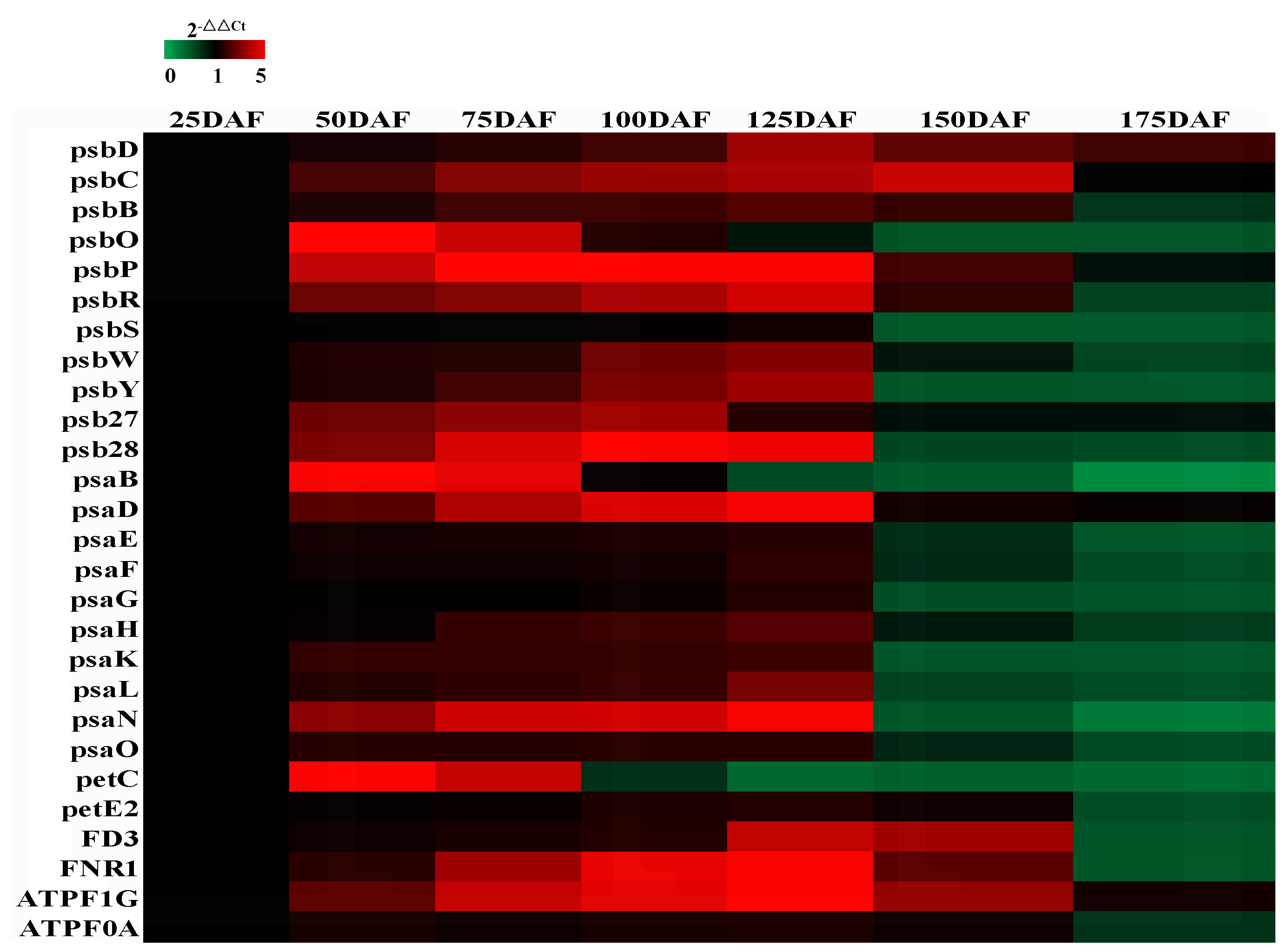

Supplement: Supplementary file 11 — Additional file 11: Figure S3. Transcript patterns of genes involved in photosynthetic light reaction in developing L. glauca fruits. [file 13068_2017_820_MOESM11_ESM.tiff]
